# Structure and ecological function of the soil microbiome affecting plant–soil feedbacks in the presence of a soil‐borne pathogen

**DOI:** 10.1111/1462-2920.14882

**Published:** 2019-12-18

**Authors:** S. Emilia Hannula, Hai‐kun Ma, Juan E. Pérez‐Jaramillo, Ana Pineda, T. Martijn Bezemer

**Affiliations:** ^1^ Department of Terrestrial Ecology Netherlands Institute of Ecology (NIOO‐KNAW) Droevendaalsesteeg 10, 6708 PB Wageningen The Netherlands; ^2^ Institute of Biology, Section Plant Ecology and Phytochemistry Leiden University P.O. Box 9505, 2300 RA Leiden The Netherlands; ^3^ Department of Microbial Ecology Netherlands Institute of Ecology (NIOO‐KNAW) Droevendaalsesteeg 10, 6708 PB Wageningen The Netherlands

## Abstract

Interactions between plants and soil microbes are important for plant growth and resistance. Through plant–soil‐feedbacks, growth of a plant is influenced by the previous plant that was growing in the same soil. We performed a plant–soil feedback study with 37 grass, forb and legume species, to condition the soil and then tested the effects of plant‐induced changes in soil microbiomes on the growth of the commercially important cut‐flower Chrysanthemum in presence and absence of a pathogen. We analysed the fungal and bacterial communities in these soils using next‐generation sequencing and examined their relationship with plant growth in inoculated soils with or without the root pathogen, *Pythium ultimum*. We show that a large part of the soil microbiome is plant species‐specific while a smaller part is conserved at the plant family level. We further identified clusters of plant species creating plant growth promoting microbiomes that suppress concomitantly plant pathogens. Especially soil inocula with higher relative abundances of arbuscular mycorrhizal fungi caused positive effects on the Chrysanthemum growth when exposed to the pathogen. We conclude that plants differ greatly in how they influence the soil microbiome and that plant growth and protection against pathogens is associated with a complex soil microbial community.

## Introduction

The interactions between plants and soil microbes are important drivers of ecosystem functions and plant community structure and diversity (Reynolds *et al*., 2003). Soil microbes can help plants with nutrient acquisition (van der Heijden *et al*., 2006; Yang *et al*., 2009), via production or regulation of plant hormones (Kim *et al*., 2011) and by protecting plants against pathogens and other stressors (Berendsen *et al*., 2012). On the other hand, soil pathogens are thought to drive succession by negatively influencing the performance of certain plant species or groups and consequently influencing the plant community turnover (Bever *et al*., 2012). The abundance and composition of microbes in the soil, in turn, is influenced by the plant that grows in the soil. This leads to plant–soil feedbacks (PSFs) where one plant can influence the growth of another plant via the impact of the first one on the soil (van der Putten *et al*., 2013). Such feedbacks may differ between plant families and among grasses, forbs and leguminous plants (Bezemer *et al*., 2006) and between nutrient‐acquisition strategies (Teste *et al*., 2017). For example, legumes fix nitrogen in association with rhizobia and consequently may increase nutrient availability for other plants, resulting in positive PSF effects (Tilman *et al*., 1997). However, growth by legumes can also alter the soil microbiome in ways that it has negative effects on other plants with different nutrient‐acquisition strategies such as species relying on arbuscular mycorrhizal fungi (AMF; Wubs and Bezemer, 2016; Teste *et al*., 2017). Via their effects on the soil, grasses generally have positive effects on the growth of forbs (Ma *et al*., 2017), but the mechanisms for this effect are still largely unknown. Both soil‐borne pathogens and mutualists such as AMF vary in their host specificity from generalist forming associations with all plants to highly specific associations (Barrett and Heil, 2012; Horn *et al*., 2017). It is uncertain if more closely related species exhibit more negative PSFs through actions of soil microbes (Bever *et al*., 2010; Cortois *et al*., 2016). Currently, little is known about how individual plant species change the soil microbiome by growing in the soil, and how this feeds back to the growth of the following plant and, for example, the ability to defend itself from pathogens.

The soil microbiome, and especially the fungal part of the microbiome (‘mycobiome’) can roughly be divided into three functional categories (mutualists, pathogens and saprotrophs) based on the functions they provide to the plant (Nguyen *et al*., 2016; van der Putten *et al*., 2016). The net outcome for plant growth will depend on antagonistic (plant pathogens) and synergistic (mutualists such as AMF, decomposers) interactions within the soil microbiome, and changes in the relative abundance of the microbial species belonging to these functional groups can greatly influence plant growth or plant health (van der Putten *et al*., 2016; Hannula *et al*., 2017). Plants that grow in a soil with a common microbiome can alter the relative abundance of mutualists, pathogens and saprotrophs in that soil, and this change can depend on the identity and taxonomy of that plant (Fitzpatrick *et al*., 2018). By monitoring the PSF‐effects of these conditioning species on other plants, we can relate these changes in the soil microbiome to plant growth or plant resistance against attack by, for example, pathogens.

An important challenge in PSF research is to use plants to steer soil microbiomes so that they improve the growth and resistance to pests or pathogens of crops (Badri *et al*., 2013; Pineda *et al*., 2017; Elhady *et al*., 2018). Plant root exudates that differ greatly between plant species play a dominant role in shaping the rhizosphere and eventually the soil microbiome (Bais *et al*., 2006; Hu *et al*., 2018). A recent study (Fitzpatrick *et al*., 2018) addressed how 30 angiosperm species changed their rhizosphere microbiomes and how this has potential consequences for PSFs. The authors addressed the role of abiotic stress on shaping the PSF responses but did not look into microbial taxa or functional guilds protecting against biotic stressors such as pathogens. Furthermore, the effect of the rhizosphere microbiome of the first plant on the growth of the following plant was larger than the effects caused by its endosphere microbiome (Fitzpatrick *et al*., 2018). Currently, it is not yet possible to predict which plants and which microbes cause positive feedbacks and whether this is conserved at higher organizational levels such as plant families or differs between groups of plants such as grasses, legumes and other forbs.

The general objective of this study is to unravel how the community structure and diversity of soil microbes is determined by the plant species that grows in the soil, and how this relates to PSF effects on growth of the following plant under pathogen pressure. Furthermore, we examine if these effects are conserved at the level of plant family or vary among broad groups of plants, such as among grasses, legumes and other forbs. In this study, we focus on PSF effects on Chrysanthemum, an important ornamental crop. We analysed the fungal and bacterial soil communities after growing 37 plant species individually in the soil and related this to the growth of Chrysanthemum in the feedback phase in inoculated soils (Ma *et al*. 2017). During the feedback phase, we added a soil‐borne oomycete pathogen, *Pythium ultimum* to a subset of plants, to examine the relationship between microbiome composition and the ability of the plant to grow in the presence of a root pathogen. *P. ultimum* causes root rot to a wide range of plants including Chrysanthemum (Lévesque *et al*., 2010) and several studies have shown that soil‐borne microbes can suppress its effects on the plant (van Os and van Ginkel, 2001; Yu *et al*., 2015). The nature of interactions between plants and soil biota will likely depend on plant characteristics such as functional traits and growth form (functional groups). We hypothesized that grasses, forbs and legumes (i.e. plants belonging to different functional groups) would differ in the community structure of fungi and bacteria in the soil. We further hypothesized that plant species that are related to each other (i.e. belonging to the same family) will select for more similar microbiomes. We previously reported that grasses have in general positive PSF effects on Chrysanthemum (Ma *et al*., 2017), while forbs and legumes have negative effects. We hypothesized that this is related to an increase in the relative abundance of microbes in the soil microbiome that induce resistance in the plant against pathogens or that act as antagonists, and in the promotion of relative abundance of beneficial bacteria and fungi such as AMF.

## Results

### *Number of reads and unique OTUs*

The 16S amplicon sequencing yielded 3.4 million high‐quality, nonchimeric sequences across all samples, with a median of 14 536 (range 1276–147 583 sequences per sample). We detected that the diversity of bacteria was strongly affected by number of reads obtained per sample and decided thus to rarefy the data to 1276 reads which was a compromise between loosing samples and loosing part of the data (Supporting Information Fig. S3). We further analysed the effects of treatments (plant species and plant group) for the datasets that were rarified to different numbers to ensure our conclusions are not based on technical artefacts (Supporting Information Fig. S3). Sequencing of the ITS2 region yielded a total of 1.2 million high‐quality fungal sequences, with a median of 6052 (range 858–19 657). The fungal data were not rarified, and rarefaction curves indicated that the number of reads did not significantly influence diversity or community structure, ensured us that sufficient sequencing depth was reached (Supporting Information Fig. S4). There were 11 306 bacterial and 3064 fungal OTUs identified in the dataset. After filtering out singletons (i.e. OTUs present only once in each category) and rare OTUs (present in less than 5 samples), we detected that 129 bacterial and 109 fungal OTUs were present in the soils conditioned by all the plants forming the core microbiome of the soils. A total of 1180 bacterial and 768 fungal OTUs were specific to certain conditioning plants. *Galium verum* had the smallest number of unique bacterial OTUs (13) but the highest number of unique fungal OTUs (478). *Matricaria recutica* had the largest number of unique bacterial OTUs (64) while *Lotus corniculatus* had the lowest number of fungal OTUs (9; Supporting Information Fig. S5). The number of unique bacterial OTUs found in the soils conditioned by at least two plant species within a plant group was 218 for forbs, 35 for grasses and 31 for legumes. Similarly, the number of unique fungal OTUs in soils conditioned by at least two plant species within a plant group was 195 for forbs, 1 for grasses and 18 for legumes (Supporting Information Fig. S5).

### *Effect of grasses, forbs and legumes on bacterial and fungal communities*

**Figure 1:**
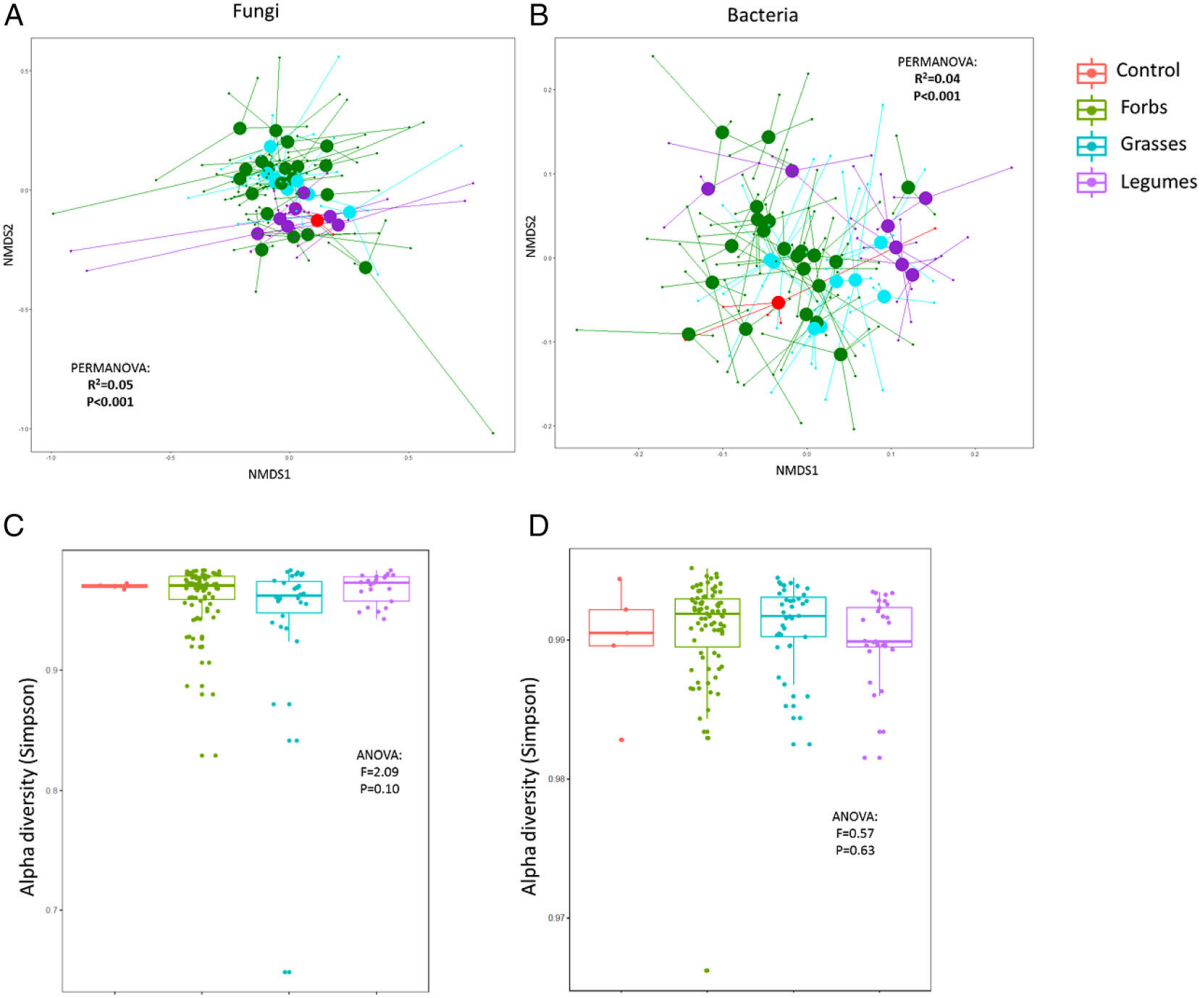
The community structure (A and B) and Simpson diversity (C and D) for bacteria (B and D) and fungi (A and C) per plant group and in the no‐plant control. No‐plant control soils are coloured red, forbs green, grasses turquoise and legumes purple. In (A) and (B) centroids are shown as large dots and lines connect the individual samples to the centroids. In (C) and (D) Tukey box‐and‐whisker plots show median diversity, 90 percentile quartiles and range while the dots depicts individual samples. Significance from a PERMANOVA test for community structure and an ANOVA for diversity are also presented in the figure. For further statistical tests, see Table 1.

There was a significant effect of plant group (i.e. forbs, grasses and legumes) on the community composition of both soil fungi and bacteria (Table 1, Fig. 1) while no effect of plant group on the α‐diversity of bacteria or fungi was detected (Table 1). Plant group (excluding the inocula belonging to the no‐plant treatment) explained around 5% of the variation in the bacterial community and 10% of the variation in fungal community structure. The fungal phyla that were significantly affected by plant group were Ascomycota, Basidiomycota and Mucoromycota, while for bacteria the *Actinobacteria*, *Planctomycetes* and subphyla *Alphaproteobacteria* and *Deltaproteobacteria* were significantly affected (Table 2, Supporting Information Fig. S6). On the level of classes, there were 9 (out of 75) bacterial classes and 5 (out of 15) fungal orders that were significantly less or more abundant in one of the plant groups than in others (Table 3). Most notably, there were relatively more AMF (Glomeromycotina) in soils in which grasses and forbs had grown than in soils of legumes, while the relative abundance of the phylum Ascomycota was higher in soils conditioned by legumes than in forb and grass soils (Supporting Information Fig. S6). Furthermore, there were slightly more Basidiomycota and especially Pucciniomycetes in soils in which grasses were grown compared to soils from legumes or forbs. The bacterial phyla *Actinobacteria* and *Planctomycetes* and the subphylum *Alphaproteobacteria* were more abundant in soils in which legumes had grown than in soils in which grasses and forbs were grown and the subphylum Deltaproteobacteria was most common in soils in which forbs had grown (Supporting Information Fig. S6).

**Table 1:** The effects of plant group (grass, forb or legume), family, species and phylogenetic distance to Chrysanthemum on Simpson (alpha) diversity and community structure (beta diversity) of bacteria and fungi; and the relationship between diversity and community structure of bacteria and fungi and Chrysanthemum biomass of plants in the control treatment without pathogen and plants exposed to *Pythium ultimum*, and their ratio.

	Bacteria				Fungi			
	α‐Diversity		Community structure		α‐Diversity		Community structure	
	*F*	*p*	*R*	*p*	*F*	*p*	*R*	*p*
Plant group (grass‐forb‐legume)	0.576	0.632	**0.035**	**0.001**	2.068	0.108	**0.115**	**0.013**
Plant species	**1.583**	**0.034**	**0.292**	**0.001**	0.851	0.706	**0.317**	**0.001**
Plant family	0.805	0.624	**0.108**	**0.002**	0.809	0.621	**0.165**	**0.001**
Phylogenetic distance	0.049	0.825	0.007	0.054	2.421	0.122	**0.024**	**0.003**
Total DW plants	0.175	0.677	**0.009**	**0.002**	0.055	0.815	**0.012**	**0.017**
DW plants infected with *Pythium*	0.628	0.429	**0.009**	**0.006**	0.477	0.491	**0.013**	**0.008**
Relative DW plants	0.066	0.797	0.007	0.310	0.810	0.370	0.007	0.436

Alpha‐diversity was measured with the Simpson index and treatments effects evaluated using linear models. Effects on community structure were evaluated using PERMANOVA.

**Table 2:** The effects of plant species and plant group (grass, forb or legume)on the abundances of fungal, archaeal and bacterial phyla and the relationship between the abundance of these phyla and the effect of conditioning plant on the relative growth of chrysanthemum under conditions without versus with the pathogen.

	Plant species (df 36,105)		Plant group (df 3,138)		Relative plant growth (without *Pythium*/with *Pythium*; df 1,134)		Relative plant growth × plant group (df 5,130)		Plant biomass with *Pythium* (df 3,132)		Plant biomass with *Pythium* × plant group (df 5,130)		Plant biomass without *Pythium* (df 1,134)		Plant biomass without *Pythium* × plant group (df 5,130)	
	*F*	*p*	*F*	*p*	*F*	*p*	*F*	*p*	*F*	*p*	*F*	*p*	*F*	*p*	*F*	*p*
Ascomycota	**2.053**	**0.003**	**8.474**	**< 0.001**	1.434	0.233	2.620	0.077	**11.242**	**0.001**	1.392	0.252	**4.243**	**0.041**	1.463	0.235
Basidiomycota	**1.904**	**0.007**	**8.117**	**0.005**	0.059	0.808	1.980	0.142	**4.850**	**0.029**	0.585	0.559	3.118	0.080	0.623	0.538
Chytridiomycota	0.825	0.441	1.203	0.237	0.887	0.348	1.488	0.230	0.193	0.662	0.577	0.563	0.179	0.673	0.153	0.858
Mucoromycota	**4.759**	**0.010**	**1.579**	**0.041**	2.186	0.142	0.541	0.584	3.099	0.081	0.432	0.650	0.498	0.482	0.790	0.456
Archaea	1.082	0.368	0.403	0.669	0.846	0.359	0.468	0.627	0.045	0.833	**3.144**	**0.046**	0.292	0.865	1.490	0.229
*Acidobacteria*	0.881	0.661	2.304	0.104	**5.799**	**0.017**	0.674	0.511	0.412	0.522	0.953	0.388	3.183	0.076	0.528	0.591
*Actinobacteria*	**2.015**	**0.003**	**9.013**	**< 0.001**	0.803	0.372	0.536	0.586	1.873	0.173	0.466	0.628	2.473	0.118	0.916	0.402
*Armatimonadetes*	0.996	0.487	2.584	0.079	0.032	0.858	1.151	0.319	0.618	0.433	0.213	0.809	0.620	0.432	0.507	0.604
*Bacteroidetes*	0.928	0.590	0.442	0.644	2.049	0.154	0.382	0.683	**4.880**	**0.029**	0.310	0.734	7.832	0.006	0.109	0.897
*WPS2*	1.127	0.312	0.287	0.751	0.474	0.492	1.102	0.335	0.193	0.662	0.411	0.664	0.751	0.388	2.156	0.120
*Chlamydiae*	**1.764**	**0.013**	0.468	0.627	0.565	0.453	0.146	0.864	0.906	0.343	0.138	0.871	1.671	0.198	0.003	0.997
*Chloroflexi*	**2.761**	**< 0.001**	0.892	0.412	0.492	0.484	0.766	0.467	1.695	0.195	0.143	0.867	0.441	0.508	0.228	0.796
*Firmicutes*	**2.063**	**0.002**	1.166	0.315	2.894	0.091	0.471	0.625	0.109	0.742	0.123	0.885	1.797	0.182	0.008	0.992
*Gemmatimonadetes*	**1.651**	**0.025**	2.714	0.070	0.615	0.543	0.859	0.426	0.508	0.477	0.055	0.947	0.643	0.424	0.545	0.947
*Planctomycetes*	**1.570**	**0.039**	**9.903**	**< 0.001**	0.025	0.874	1.938	0.148	1.121	0.291	0.816	0.444	0.494	0.483	0.416	0.660
*Alphaproteobacteria*	**1.596**	**0.033**	**3.325**	**0.039**	0.099	0.754	0.224	0.800	0.068	0.795	0.369	0.672	0.169	0.682	0.172	0.842
*Betaproteobacteria*	**3.368**	**< 0.001**	1.946	0.147	0.200	0.655	0.792	0.455	**14.531**	**< 0.001**	0.181	0.167	**13.210**	**< 0.001**	0.926	0.926
*Deltaproteobacteria*	**2.816**	**< 0.001**	**10.050**	**< 0.001**	0.008	0.929	0.851	0.429	1.812	0.180	2.464	0.089	1.340	0.249	1.620	0.202
*Gammaproteobacteria*	1.469	0.066	1.054	0.351	0.000	0.982	1.210	0.301	0.486	0.487	0.311	0.733	1.445	0.231	1.162	0.316
*Verrucomicrobia*	1.262	0.179	0.107	0.899	0.443	0.507	0.657	0.520	0.416	0.520	0.345	0.709	0.369	0.545	0.026	0.974

Significant values derived from linear models are marked in bold.

**Table 3:** The effects of plant species and plant group (grass, forb or legume)on the abundances of fungal classes and the relationship between the abundance of these phyla and the effect of conditioning plant on the relative growth of chrysanthemum under conditions without versus with the pathogen.

	Plant species (df 36,105)		Plant group (df 3,138)		Relative plant growth (without *Pythium*/with *Pythium*; df 1,134)		Relative plant growth × plant group (df 5,130)		Plant biomass with *Pythium* (df 3,132)		Plant biomass with *Pythium* × plant group (df 5,130)		Plant biomass without *Pythium* (df 1,134)		Plant biomass without *Pythium* × plant group (df 5,130)	
	*F*	*p*	*F*	*p*	*F*	*p*	*F*	*p*	*F*	*p*	*F*	*p*	*F*	*p*	*F*	*p*
Dothideomycetes	**1.645**	**0.029**	0.897	0.410	0.061	0.805	1.297	0.277	0.000	0.994	0.391	0.677	0.205	0.651	1.483	0.231
Eurotiomycetes	1.349	0.127	**4.991**	**0.008**	0.316	0.575	0.147	0.864	**8.622**	**0.004**	0.769	0.466	3.818	0.053	0.269	0.764
Leotiomycetes	**1.786**	**0.014**	2.443	0.091	0.040	0.842	1.252	0.289	0.419	0.518	1.949	0.147	0.265	0.608	3.021	0.052
Orbiliomycetes	1.241	0.202	0.939	0.394	0.489	0.486	0.463	0.531	0.992	0.321	2.107	0.126	3.957	**0.049**	**4.696**	**0.011**
Pezizomycotina	1.063	0.396	0.716	0.491	0.040	0.841	1.130	0.326	0.105	0.747	1.564	0.213	0.011	0.918	**6.385**	**0.002**
Saccharomycetes	1.460	0.075	0.797	0.453	0.045	0.832	1.265	0.286	0.004	0.947	0.839	0.435	0.098	0.755	0.393	0.676
Sordariomycetes	**1.694**	**0.022**	1.322	0.270	0.441	0.508	0.247	0.782	1.798	0.182	0.337	0.715	0.737	0.392	0.032	0.969
Agaricomycetes	0.963	0.537	0.878	0.418	0.201	0.655	2.070	0.130	0.316	0.575	1.602	0.206	0.232	0.597	2.819	0.063
Microbotryomycetes	0.628	0.941	0.349	0.706	0.289	0.592	0.075	0.928	0.460	0.499	1.976	0.143	0.340	0.561	1.734	0.181
Pucciniomycetes	**4.123**	**< 0.001**	**20.313**	**< 0.001**	0.428	0.514	**3.946**	**0.022**	0.863	0.004	0.004	0.996	6.177	**0.014**	**3.246**	**0.042**
Tremellomycetes	**2.448**	**< 0.001**	**9.212**	**< 0.001**	0.001	0.973	0.038	0.962	3.016	0.085	0.264	0.769	**4.141**	**0.044**	1.738	0.180
Chytridiomycetes	1.320	0.144	0.632	0.533	0.319	0.574	2.985	0.054	0.144	0.705	0.618	0.541	0.363	0.548	0.329	0.720
Glomeromycetes	**3.970**	**< 0.001**	**8.939**	**< 0.001**	0.981	0.324	0.335	0.716	**8.394**	**0.004**	0.537	0.586	3.180	0.077	0.471	0.626
Mortierellomycotina	0.664	0.915	0.154	0.858	1.165	0.283	1.643	0.197	0.086	0.770	0.277	0.759	0.531	0.468	0.708	0.494
Mucoromycotina	1.533	0.052	**5.079**	**0.007**	1.206	0.274	0.753	0.473	2.440	0.121	0.230	0.795	0.012	0.915	1.223	0.298

Significant values derived from linear models are marked in bold.

**Figure 2:**
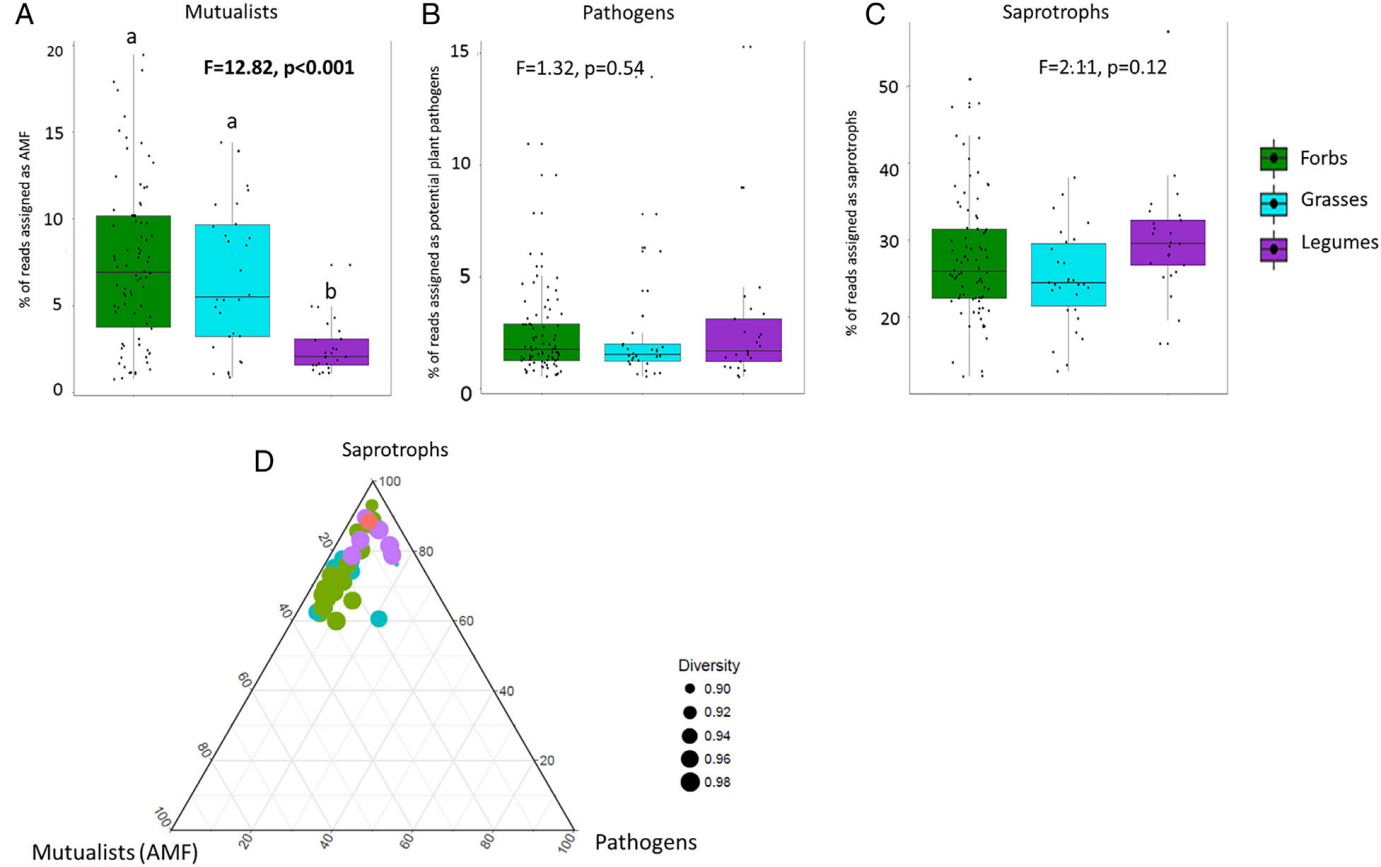
The relative abundance of fungal mutualists (A), plant pathogens (B) and saprotrophs (C) and their relationship (D) expressed per plant group. Control soils (unconditioned soils) are coloured red, forbs green, grasses turquoise and legumes purple. Tukey box‐and‐whisker plots show median diversity, 90 percentile quartiles and range while the dots depicts individual samples. Significance from an ANOVA test is also presented in (A)–(C). In the ternary plot (D), the average fungal diversity of plant species is represented by the size of the marker.

Three fungal functional guilds were significantly affected by plant group: AMF (Fig. 2A), a combined guild consisting of fungal parasites and saprotrophs, and a combined guild consisting of potential plant pathogens and saprotrophs (Supporting Information Table S2). AMF were most abundant in the soils conditioned by forbs followed by grasses while the two other guilds were more common in the soils conditioned by legumes. The guilds consisting only of potential plant pathogens and only of saprotrophs were not significantly affected by plant group (Fig. 2B and C). Ternary plots were used to investigate how broad plant groups affected the continuum of potential mutualist–pathogen–saprotroph space. This showed that grasses and forbs shifted the community towards more symbiotic microbes, while legumes shifted it towards more plant pathogenic microbes or kept it enriched in saprotrophs (Fig. 2D).

### *Plant species shaping bacterial and fungal communities*

There was a significant plant species‐specific effect on the diversity of bacteria but not fungi (Table 1, Supporting Information Fig. S8). For bacteria, the highest diversity was detected in soils in which *Arnica montana* and *Tagetes minuta* had been grown, while lowest bacterial diversity was detected in soils conditioned by *Plantago lanceolata* (Supporting Information Fig. S8). There was a strong overall effect of plant species on both fungal and bacterial community structure (Table 1) explaining 29% (bacteria) and 32% of the variation (fungi). The relative abundances of reads assigned to three fungal and nine bacterial phyla were significantly affected by plant species identity (Table 2; Supporting Information Fig. S9). Furthermore, six fungal classes were affected by plant species identity. For fungi, the phyla and classes that were affected by plant species were almost all the same as those significantly affected by plant groups but for bacteria this was not the case (Supporting Information Fig. S9). Six of the functional guilds of fungi were affected by plant species identity (see Supporting Information Table S2 and Fig. S10). The plant species effects on the relative abundance of fungal parasites and ericoid mycorrhizal fungi/dark septate endophytes were driven by a few plant species having these associations while others not. For example, ericoid mycorrhizal/dark septate endophyte fungi were enriched in soils after growth by the forbs *Matricia recutita* and *Tanacetum vulgare*, the grass *Lolium perenne* and the legumes *L. corniculatus* and *Vicia cracca*. The largest variation among plant species was detected for AMF and for saprotrophs (Supporting Information Fig. S10). This is also reflected in the difference in position of plants in the pathogen‐mutualism‐saprotrophs continuum (Fig. 2C). The relative abundance of Glomeromycetes (AMF) was highly variable among species of forbs. AMF were rare in all inocula from soils where legumes had grown, and the observed significant plant species effects on AMF were mainly caused by differences among forbs. The forbs, *Arabidopsis thaliana*, *Campanula rotundifolia*, *Capsella bursa‐pastoris*, *Rumex acetosella* and *Thymus pulegoides* had significantly lower relative abundance of AMF reads than other forbs. Similar separation as observed in the full dataset including all plant species was observed for the subsets of the species. For AMF, the field soil, which was kept in the greenhouse but not grown by plants after collection from the grassland (no‐plant treatment) also had significantly fewer AMF reads than the soils conditioned by plants on average (Fig. 2).

### *Plant family and phylogenetic distance*

We subsequently compared plant families. As all grasses and legumes belonged to one family per group (Poaceae and Fabaceae respectively), only forbs were included in the analysis. There was no significant effect of plant family on the alpha diversity of bacteria or fungi (Table 1, Supporting Information Fig. S11). However, community structure of both bacteria and fungi was significantly affected by plant family and for fungi this was also true for the phylogenetic distance to Chrysanthemum.

### *PSF effects on chrysanthemum performance*

**Figure 3:**
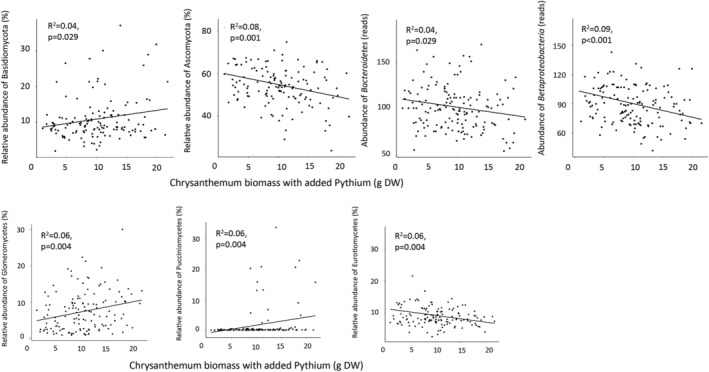
Significant correlations between the relative abundance of fungal and bacterial phyla (upper panels) and fungal classes (bottom panels) and Chrysanthemum biomass in pots with *Pythium ultimum* addition.

Alpha‐diversity of bacteria or fungi was not significantly related to growth responses of Chrysanthemum in absence or presence of *P. ultimum* (Table 1). However, community structure of both bacteria and fungi was related to the dry‐weight of the Chrysanthemum plants both with and without *P. ultimum* addition. The relative abundance of Ascomycota was negatively, and the relative abundance of Basidiomycota positively correlated with biomass of Chrysanthemum in the presence of *P. ultimum* and this did not depend on plant group (Table 2 and Fig. 3). Furthermore, the relative abundance of ascomycetes was negatively correlated with biomass of the control plants (Table 2). The relative abundance of the bacterial phylum *Bacteroidetes* and the subphylum *Betaproteobacteria* were negatively correlated with the biomass of Chrysanthemum both in the absence and presence of *P. ultimum* indicating that they had a general negative effect on the growth of the Chrysanthemum. Abundance of members of the phylum *Acidobacteria* was negatively correlated with the relative performance of Chrysanthemum (i.e. the difference between plants grown with and witout *P. ultimum* Fig. 3 and Table 2). We looked more specifically into the orders within the *Bacteroidetes*, *Betaproteobacteria* and *Acidobacteria* and detected that the negative effects on Chrysanthemum biomass were correlated with abundances of *Bacteroidales* (*r* = −0.16, FDR *p* < 0.05),* Acidobacteria* group 7 (*r* = −0.22, FDR *p* < 0.01), and Burkholderiales (*r* = −0.22, FDR *p* < 0.01).

For fungi, at class level, an increase in the relative abundance of Eurotiomycetes was negatively and the relative abundance of Glomeromycetes positively correlated with the biomass of Chrysanthemum in the presence of *P. ultimum* (Fig. 3). In absence of *P. ultimum*, Orbiliomycetes and Pucciniomycetes were positively correlated with Chrysanthemum biomass. The increase in relative abundance of both classes also differed among grasses, forbs and legumes. The relative abundances of AMF families related with plant performance in the presence of *P. ultimum* were Claroideoglomeraceae (*r* = 0.23, FDR *p* < 0.01), Archaeosporaceae (*r* = 0.19, FDR *p* < 0.05), and Paraglomeraceae (*r* = 0.23, FDR *p* < 0.01). Within the Eurotiomycetes, the family Trichocomaceae correlated negatively with the biomass of Chrysanthemum in the presence of *P. ultimum* (*r* = −0.16, FDR *p* < 0.05).

### *Core microbiome in disease and health*

**Figure 4:**
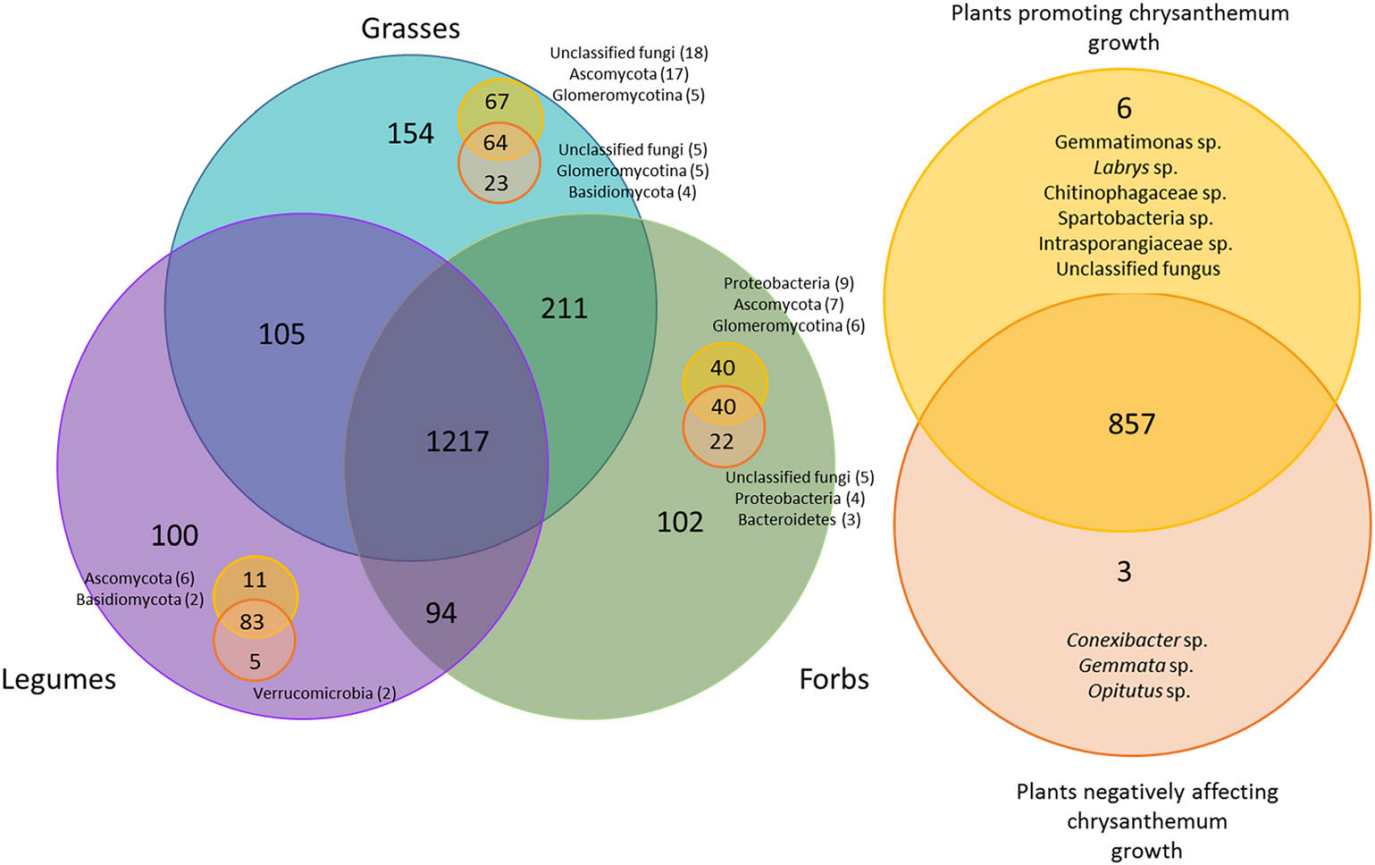
Number of shared OTUs between plant groups (grasses, forbs and legumes) and the feedback they caused to the growth of Chrysanthemum (for classification, see Supporting Information Fig. S2). Forb soils are coloured green, grasses turquoise, and legumes purple. Plants having growth promoting effect on chrysanthemum are depicted in yellow and plants with negative feedback with orange. For OTU to be present in plant group, it needed to be present in at least 20% of the samples. For the right side, OTU needed to be present with > 20% of samples in all three groups of plants. The category specific (good forbs, bad forbs, good grasses, bad grasses, good legumes, bad legumes) fungal and bacterial phyla to which the majority of OTUs belonged in the category are shown in the left panel. The genera that caused positive or negative feedbacks across plant groups are listed on the right side.

We explored the core soil microbiome shaped by major plant groups (grasses, forbs and legumes) separately for plant species showing a positive effect on Chrysanthemum growth and plants causing a negative effect on Chrysanthemum growth. A total of 1217 OTUs (56% of all OTUs) were shared between grasses, forbs and legumes, while only 100 to 154 OTUs (4%–7% of all OTUs) were specific to one of these groups (Fig. 4). Grasses had most unique OTUs (154 OTUs) and also shared most OTUs with forbs (211 OTUs). When looking across plant groups for OTUs that were only found in soils causing an increase in growth of chrysanthemum, six OTUs (that is less than 0.5% of the OTUs) were consistently selected for across groups of plants and at the same time increased plant growth. These OTUs were Gemmatimonas sp. (Gemmatimonadetes), *Labrys* sp. (Proteobacteria), Chitinophagaceae sp. (Bacteroidetes), Spartobacteria sp. (Verrucomicrobia), Intrasporangiaceae sp. (Actinobacteria), and an unclassified fungus. On the other hand, there were three OTUs that were associated with stunned growth of chrysanthemum that were also found across plant groups. A total of 857 OTUs (around 40% of all OTUs) were found across plant groups and both in soils having positive and negative effects on chrysanthemum growth. When looking per plant group at OTUs found in soils that promoted chrysanthemum growth, we detected that 44% of the OTUs unique to grasses, 39% of OTUs unique to forb and 11% of OTUs unique to legumes, were found in soils of plants causing a positive effect on chrysanthemum but not in soils of plants causing negative effects (Fig. 4). The major phyla these beneficial plant group specific OTUs could be assigned to were Ascomycota (for grasses, legumes and forbs), Glomeromycotina (grasses and forbs), Basidiomycota (legumes) and Proteobacteria (forbs). Fewer OTUs were associated solely with plants causing a negative feedback effect on chrysanthemum. For forbs 22%, for grasses 15% and for legumes 5% were unique to those groups and were found only in plants creating a negative soil effects.

### *Clusters causing positive and negative feedback effects*

**Figure 5:**
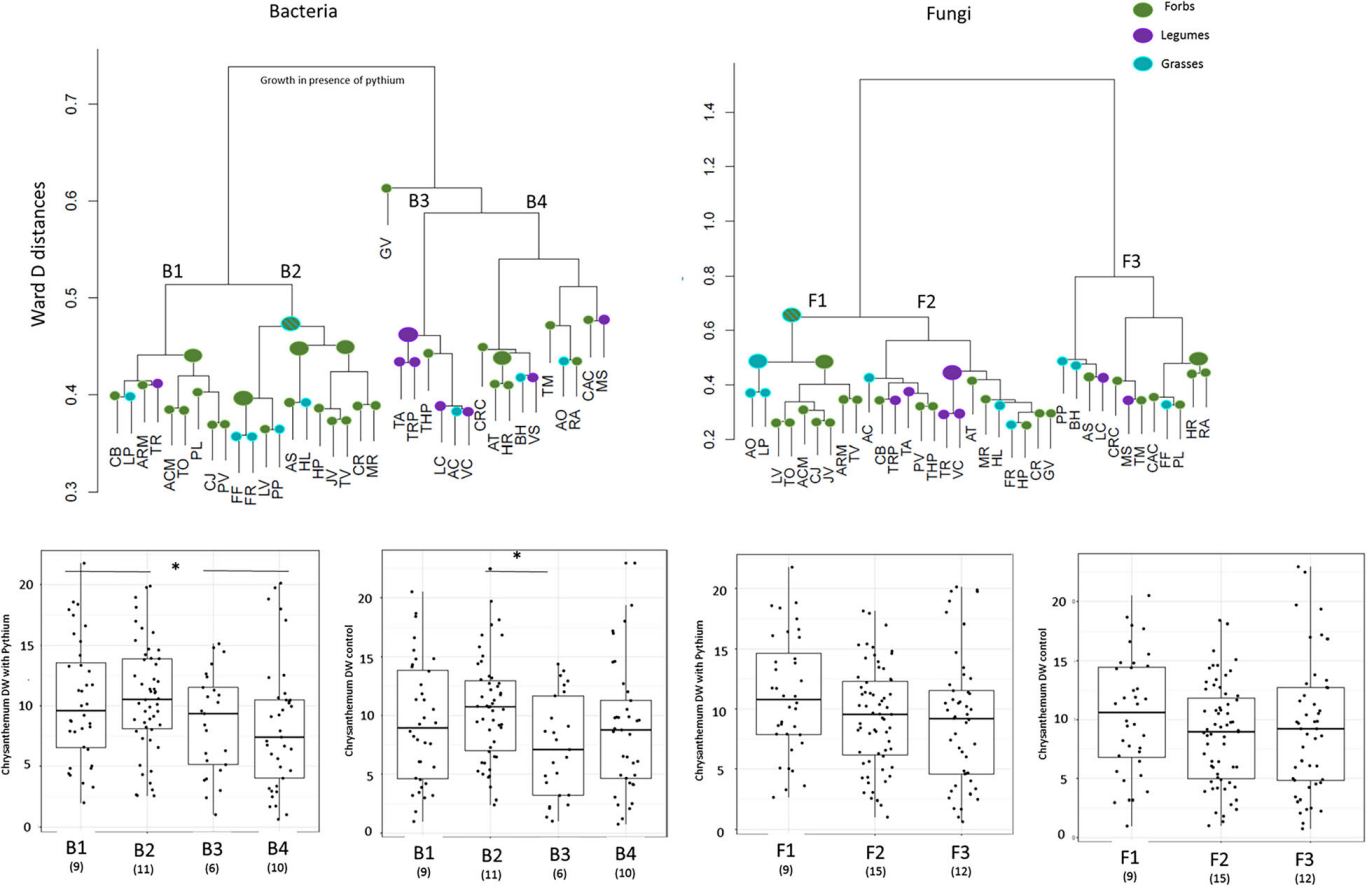
Ward's distance dendrograms for bacterial and fungal communities depicting the distance between individual plant species. Forbs are coloured green, grasses turquoise and legumes purple. Higher level of clustering, if conserved, is marked with larger coloured nodes. If two groups of plants are present in a cluster, the node is striped. The plants are divided into four bacterial and three fungal community types based on the clustering. The performance of each cluster in the feedback phase with and without *Pythium ultimum* addition is shown in the lower panels. Tukey box‐and‐whisker plots depict the median Chrysanthemum biomass of all fungal and bacterial clusters in presence and absence of added *P. ultimum*. The number of plant species in each cluster is indicated in brackets below the name of the cluster. Asterisk notes significance of GLM between clusters at the level of *p* < 0.05.

Based on a distance matrix among individual plants using Ward's distance, we detected that there was some conservation of microbiomes between plant groups but this had exceptions (Fig. 5). For example, the bacterial community of the legume *Trifolium repens* clusters closest to the bacterial community of the forb *A. montana*. We divided the plant species into clusters (B1–B4 and F1–F3) based on similarities in their bacterial (B) and fungal (F) communities using the full dataset, and further related the clusters to measured responses of Chrysanthemum growth in presence and absence of the pathogen in order to evaluate the effects of microbial community structure on plant growth. The size of the clusters was: fungal cluster F1 contained 9 plant species, cluster F2 contained 16 plant species and cluster F3 contained 13 plant species while bacterial cluster B1 included 9 plant species, B2 11 plant species, B3 6 plant species and B4 11 plant species. For bacteria, conditioning of soils with plants from clusters B1 and B2 (20 plants in total) led to significantly greater biomass of Chrysanthemum in the presence of the *Pythium* pathogen than conditioning soils with plants from clusters B3 and B4 (17 plant species; *F* = 7.091, *p* < 0.01; Fig. 4). The groups B1 and B2 contain one legume species (*T. repens*) but were dominated by grasses and forbs. However, there were no significant interactions between plant group (grass, forb or legume) and cluster (*F* = 2.177, *p* > 0.05). Furthermore, there were differences in the feedback measured as biomass of Chrysanthemum without addition of *P. ultimum* between plants clustered based on their bacterial communities. The plants selecting for bacterial community type B2 (11 plant species) caused significantly higher growth of Chrysanthemum than plant species with bacterial community B3 (6 plants; *F* = 3.875, *p* < 0.05). Division of fungal groups based on taxonomy was not significantly related to plant growth although the group F1 seemed to have species with more positive feedback on Chrysanthemum biomass both in absence and presence of *P. ultimum*. The bacterial phyla that were relatively more abundant in community type B3 were *Actinobacteria*, *Planctomycetes*, and *Alphaproteobacteria* (Supporting Information Fig. S12). Cluster B1 contained most *Chloroflexi* and cluster B2 most *Verrucomicrobia*. For fungi, Glomeromycetes and Mucoromycota in general, were most commonly found in cluster F1 but also significantly more in bacterial clusters B1 and B2 (Supporting Information Fig. S12). Ascomycota were relatively more abundant in plants belonging to cluster F2 and B3. At the level of classes, there were relatively more Eurotiomycetes and least Glomeromycetes in cluster B3.

## Discussion

Biotic interactions via plant–soil‐feedbacks are important for the growth of plants and for terrestrial ecosystems as a whole (van der Putten *et al*., 2013, 2016; Bennett *et al*., 2017). Soil microbes are thought to be key drivers of PSFs both through directly affecting plant growth or defence responses, and indirectly via, for example, affecting mineralization or by acting as antagonists of plant pathogens (van der Putten *et al*., 2013; Chialva *et al*., 2018). Here, we show that we can use plants to modulate the soil microbiome but that it is difficult to predict the outcome of the feedback effect based on plant group identity or phylogenetic distance, and that the way plants shape their microbiome varies greatly between species. However, we also show that the type of plant species (i.e. grass, forb or legume) can explain part of the soil microbiome, which in some cases (like for nodule forming bacteria for legumes) overrides the effects of plant species. The proportion of variation in communities explained by these three plant groups is between 4% (bacteria) and 11% (fungi) while plant species identity explains around 30% of the variation in microbial communities. The relative abundance of some groups of bacteria and fungi in the microbiome depends on the plant species that grows in the soil, while the abundance of other groups varies among plant groups. We did not measure here the effects of previous plant through other mechanisms than changes in the microbiome. In the second phase of the experiment, however, 90% of the soil was sterilized and hence identical in terms of soil chemistry between treatments, and we thus think that effects on the chrysanthemum growth are here due to effects prompted by microbes.

The bacterial phyla we found more commonly in the soils in which legumes had been grown (*Actinobacteria*, *Planctomycetes*, and *Alphaproteobacteria*) contain species earlier found to be enriched in legume soils and that can form associations with rhizobia (Hartman *et al*., 2017; Fitzpatrick *et al*., 2018). In the soils in which forbs had grown, the *Deltaproteobacteria* were enriched. Also, for fungi the pattern was clear on the phylum‐level: forbs conditioned the soil to have more Mucoromycota while grasses caused enrichment in Basidiomycota and legumes favoured Ascomycota. However, there was large variation in these abundances across plant species and as most of the microbial functions are not conserved at the phylum level (Kaiser *et al*., 2016), we can only speculate on the consequences of these observed patterns. An important finding in our study is that we could not predict the direction of the PSF solely from the plant group or family, even though soils from grasses tended to have more positive feedbacks than soils conditioned by forbs and legumes (Ma *et al*., 2017).

Several studies have shown that plants have species‐specific effects on the diversity and structure of the soil microbial community (Bulgarelli *et al*., 2013; Naylor *et al*., 2017; Fitzpatrick *et al*., 2018). However, often these plant specific effects are not predictable. Host phylogeny, for example, has been identified as a factor that can explain the composition of microbes in the endosphere in a predictable manner, but not for rhizosphere bacterial communities (Fitzpatrick *et al*., 2018; Leff *et al*., 2018). The rhizosphere microbiome is also not responding strongly to a signal of plant traits (Leff *et al*., 2018) and these plant traits seem to play a larger role in modulating PSFs in (more natural) mixed communities than in monocultures (Baxendale *et al*., 2014). Here we show that plant species strongly influence the composition of their soil microbiome and that this leads to a PSF effect on the growth of another plant species both in absence and in presence of a root‐pathogen. The ability to promote growth and suppress the root‐pathogen is not, however, strongly conserved in broad or narrow plant groups or in phylogeny. We did not include the soil abiotic component here and grew the plants in nutrient rich environment, but in future studies, it would be interesting to investigate the effects of abiotic and biotic components separately and in combination.

In our study with 37 plant species, certain plant species changed the soil microbiome in a specific way and inoculation with these soils enhanced the growth of a specific following plant (Fig. 5). Based on these findings we propose that within the context of PSFs, plants should not be divided into broad groups or growth forms or based on phylogenetic relatedness, but based on the microbiome that they create. In our study, plants were divided into four clusters based on the bacterial community they selected and into three clusters based on their mycobiome. Furthermore, we could show that inoculation with soil of one of these bacterial clusters increased the performance of the succeeding plant, Chrysanthemum, and at the same time, modulated the fungal community. This leads to the hypothesis that part of the changes observed in the bacterial and fungal communities are not directly due to plant growth but due to biotic interactions belowground. Especially, our results suggest that the presence and relative abundance of AMF may act as a modulator of bacterial community type and via this influences the performance of the following (nonmycorrhizal) plant indirectly (Sikes *et al*., 2010). So far, many soil microbial studies have focused on the microbiome as one entity, and further studies are needed that examine the role of interactions among groups of organisms such as bacteria and fungi within these microbiomes. Other possible explanations for the nonphylogenetic signal in regulation of the soil microbiomes are for example the composition of root‐exudates which is also dependent on growth stage and nutritional status of the plant (Badri and Vivanco, 2009; Chaparro *et al*., 2014).

We adapted the concept from van der Putten and colleagues (2016) and evaluated the changes in the composition of the fungal community along the mutualist‐pathogen‐decomposer spectrum. The fungal functional composition shifted from the nonconditioned soil treatment in most cases towards a mutualist‐, in our case AMF, rich community (Fig. 2). Forbs and individual species of grasses shifted the community towards more beneficial fungi while legumes had more neutral or even negative effects through accumulation of pathogens. However, as fungal pathogens can be specific to certain genera of plants (Cortois *et al*., 2016) and because of uncertainties in using FunGuild to predict plant pathogens correctly, their role in the feedbacks is unclear. The patchy distribution of AMF between plant species has been shown to affect grassland productivity (De Deyn *et al*., 2011). We now show that promotion of AMF in the soil by certain plants can cause a positive feedback on the growth of the following plant when it encounters root pathogens. This is in line with previous studies on the benefits of AMF in nutrient‐rich conditions. Newsham and colleagues (1995) hypothesized that AM function is based on root architecture and that plants with simple rooting systems use mycorrhizae for nutrient uptake while plants with more complex root systems are more susceptible to root pathogens and thus need AMF for protection against pathogens. The function of AMF is dependent also on the identity of the fungus (Lewandowski *et al*., 2013). Gigasporales are more effective in enhancing nutrient levels in plants while Glomeraceae better protects plants from root pathogens (Maherali and Klironomos, 2007). Our results also show that abundance of Claroideoglomeraceae, Archaeosporaceae and Paraglomeraceae are associated to the positive feedback effects on the growth of Chrysanthemum. In the current study, we do not address how much of the microbiome that is changed by plant‐conditioning remains present in the soils during the growth of the following plant. We know from our own work (unpublished data) that this cultivar of Chrysanthemum is not colonized by AMF (colonization percentages ranging from 0% to 1% and Glomeromycotina are not detected in root samples) so the positive effect of the AMF that we observed is likely to be indirect. There is recent evidence that the presence of AMF can change the composition of the soil microbiome through both positive and negative interactions and that soil microbes can, in turn, suppress the AMF (Svenningsen *et al*., 2018). Further studies should investigate which part of the soil microbiome from the conditioning phase is found inside the roots and in the soils in the feedback phase. However, there is evidence that the initial soil microbiome has the largest effect on plant performance (Wei *et al*., 2019).

Other groups that showed a positive association with plant growth were Orbiliomycetes and Pucciniomycetes. The positive effects of Orbiliomycetes can be indirect as some of them are identified as nematode‐trapping fungi (Yang *et al*., 2007). Furthermore, nontarget pathogens for Chrysanthemum from the class Pucciniomycetes that were enriched by grasses positively correlated with Chrysanthemum growth, which is in line with the expectation that nontarget pathogens can have positive effects on plant growth (Cortois *et al*., 2016). *Betaproteobacteria* and *Bacteroidetes* were related to negative feedback effects on plant growth both in the presence and absence of *P. ultimum* while *Acidobacteria* were negatively related to the relative growth of Chrysanthemum. In earlier studies it has been hypothesized that bacteria belonging to the *Bacteroidetes* have a positive effect on plant growth (Pérez‐Jaramillo *et al*., 2018) but here we show that more *Bacteroidetes* in the conditioning phase leads to growth reduction of the following plant. This could be due to different species and communities within *Bacteroidetes* eliciting the effects in different systems. *Betaproteobacteria* and especially the order *Burkholderiales* were negatively correlated with plant performance, potentially due to the pathogenicity of the members of this order (Eberl and Vandamme, 2016) or due to indirect effects through affecting the fungal community (de Boer *et al*., 2015) even though there are also many plant growth promoting strains within *Bulkholderiales*. Increased amounts of Eurotiomycetes, and more specifically Aspergilli and Penicilli, caused negative feedback effects on the Chrysanthemum growth only in the presence of *P. ultimum*. Aspergilli are opportunistic pathogens known to cause secondary infections after a primary infector has done the initial damage (Perrone *et al*., 2007) in this case aggravating the effects of *Pythium* on the growth of Chrysanthemum. An earlier study using compost as a substrate (and thus excluding the effects of AMF) showed that Actinobacteria, Acidobacteria gp14 and Cystobasidiomycetes were the groups of bacteria and fungi best able to suppress *P. ultimum* in cucumber (Yu *et al*., 2015). We did not find that these same groups suppressed *Pythium* in our system, which is probably due to our selection of the active rhizosphere microbiome and not adding organic substrates.

Each broad group of plants (grasses, forbs and legumes) had their own subset of microbes that were found only among those plants (Fig. 4). The core microbiome of the soil was, however, much larger and most OTUs were found not‐consistently be selected by plants belonging to the same group. Furthermore, there were specific taxa causing negative and positive feedback effects on the growth of following plant for each plant group and very few taxa (9) showed consistently positive or negative effects across plant types. However, it is possible that particular microbial species are consistently helping the plant to perform better, but that such effects are neutralized by the negative effects of other microorganisms and that this prevented us from detecting these interactions. Our study highlights that keystone microbial taxa (Banerjee *et al*., 2018) that can cause positive and negative feedbacks on plants may vary greatly among different groups of plants and among plant species. Future studies should focus more on functional responses of communities and examine separate mechanisms for different plant groups rather than searching for individual microbial OTUs showing consistent effects across plant groups.

## Experimental procedures

### *Plant material*

We used 36 wild plant species that are native to temperate grasslands in the Netherlands and one domesticated crop (*T. minuta*) to condition the soil. In total, there were 9 grasses, 21 forbs and 7 legumes (Supporting Information Fig. S1). Some of the selected plant species are known to have antagonistic effects on soil‐borne diseases or have promotion effects on beneficial microbes (Supporting Information Table S1) while almost nothing is known on the level at which these traits are conserved. We obtained seeds of the wild species from a wild plant seed supplier (Cruydt‐Hoeck, Assen, Netherlands) and the seeds of *T. minuta* were obtained from a garden plant seed supplier (Vreeken seeds, Dordrecht, Netherlands).

### *Experimental set‐up*

The experimental set‐up is presented in Supporting Information Fig. S1. The experiment consisted of two phases, in the first phase, the conditioning phase, we grew the plant species individually to condition soil (Ma *et al*., 2017). In the second phase, the test phase, we measured the effect of the changes in microbiomes on the performance of Chrysanthemum with and without addition of *P. ultimum*.

### *Phase I: the conditioning phase*

The experimental part describing plant growth has been described previously (Ma *et al*., 2017). Briefly, we used soil from a semi‐natural grassland where the agricultural activities were ceased in 1995 (Mossel, Ede, Netherlands, 52°04′ N, 5°45′ E), and homogenized and sieved it (1 cm mesh size) to remove coarse fragments and all macro‐arthropods. The soil was characterized as holtpodzol, sandy loam (94% sand, 4% silt, 2% clay, ~5% organic matter, 5.2 pH, 1060 mg kg^−1^ N, 75 P_2_O_5_ mg 100 g^−1^ P, 1.9 mmol+ kg^−1^ K). The soil was sterilized using gamma irradiation (> 25 K Grey gamma irradiation, Isotron, Ede, Netherlands) and pots (13 cm × 13 cm × 13 cm) were filled with a total of 1.6 kg of a (1:1) mixture of sterilized field soil and live field soil. There was no history of chrysanthemum growth or detected incidences of *Pythium* root‐rot in these soils. We grew four 1‐week old seedlings in monocultures in each pot. Each treatment included five replicates with one pot per replicate. One treatment consisted of pots filled with soil but without plants ‘no‐plant treatment’. In total, the conditioning phase comprised of 190 pots (monocultures of 37 plant species and pots without plants × 5 replicates). All pots were placed randomly in a greenhouse with 70% relative humidity, 16 h 21°C (day) and 8 h 16°C (night). Natural daylight was supplemented by 400 W metal halide lamps (225 μmol s^−1^ m^−2^ photosynthetically active radiation, one lamp per 1.5 m^2^). The pots were watered regularly.

Ten weeks later, the aboveground parts of the plants and the majority of roots were removed from the soil. Finer roots were left in the soil. The soil from each pot was homogenized, and sampled for molecular analysis. These molecular samples were stored at −80°C in Eppendorf tubes and the rest of the soil from each pot was stored in a plastic bag at 4°C (1 bag for each pot) until used in the test phase. During both phases, the same codes were used so that each pot and molecular sample from that pot could be directly compared.

### *Phase II: the test phase*

In the test phase, pots (11 cm × 11 cm × 12 cm; length × wide × height) were filled with 10% soil inoculum (plant species‐specific conditioned soil or no‐plant soil), homogenized and mixed by hand with 90% sterilized field soil (Ma *et al*. 2017; Supporting Information Fig. S1). This approach minimizes the effect of potential changes in soil abiotic characteristics as 90% of the background soil is the same between treatments. The focal plant in our study was Dendranthema X grandiflora (Ramat.) Kitam. cv. Grand Pink (Chrysanthemum, syn. Chrysanthemum X morifolium (Ramat.) Hemsl., Asteraceae). We planted these Chrysanthemum cuttings (without roots) in each pot for rooting for 10 days. Five days after rooting, 3 ml of the oospore suspension (ca. 355 000 oospores of *P. ultimum*) was added to the Chrysanthemums that were allocated to the disease treatment (van der Wurff *et al*., 2011). Plants were fertilized following common Chrysanthemum grower practice: half‐strength Hoagland nutrient solution for the first 2 weeks, single strength Hoagland solution during the following 2 weeks, and increased strength of Hoagland solution (1.6 mS cm^−1^ electrical conductivity), for the last 2 weeks. The plants were kept in a greenhouse compartment with conditions presented above. Six weeks after pathogen inoculation, all plants were harvested by clipping at soil level and roots were rinsed to remove the soil. Shoot and root biomass were oven‐dried (60°C for 3 days) and weighed (Ma *et al*., 2017). We measured Chrysanthemum biomass separately both in the pots without *P. ultimum* and in the *P. ultimum* inoculated pots. The difference in biomass in noninoculated pots – biomass in inoculated pots within each replicate was used to calculate the effect of inoculation on the sensitivity of plant growth to soil pathogen addition. For all plant families, we calculated phylogenetic distance to Chrysanthemum using maximum likelihood method. Plant family was used as a proxy for difference in phylogeny between all individual plant species.

### *Microbial DNA extraction and sequencing*

DNA was extracted from 0.25 g of soil using the PowerSoil DNA isolation kit (Qiagen, Hilden, Germany) following manufacturer's instructions. We amplified bacterial and fungal DNA in duplicate polymerase chain reaction (PCR) reactions using bar‐coded primers. For bacteria, the primers 515F/806R (5′‐GTGCCAGCMGCCGCGGTAA‐3′/5′‐GGACTACHVGGGTWTCTAAT‐3′) targeting the V4 region of the 16S rRNA gene were used (Caporaso *et al*., 2012) and for fungi the primers ITS4/ITS9 (5′‐TCCTCCGCTTATTGATATGC‐3′/5′‐GAACGCAGCRAAIIGYGA‐3′) targeting intergenic transcribed spacer (ITS2) region were used (Ihrmark *et al*., 2012). PCR products were purified using the Agencourt AMPure XP magnetic bead System (Beckman Coulter Life Sciences, Indianapolis, Indiana, USA). Purified PCR products were analysed with a Fragment Analyser using a Standard Sensitivity NGS Fragment Analysis kit and following manufacturer's instructions (Advanced analytical technologies GmbH, Heidelberg, Germany). Finally, the libraries were pooled and sequenced using the Illumina MiSeq PE250 platform (at Beijing Genomics Institute, Beijing, China).

### *Bioinformatic analysis*

The data for bacteria were analysed using an in‐house pipeline (De Hollander, 2017). In short, this pipeline uses VSEARCH to pair sequences, the SILVA database with SINA classification and VSEARCH again to cluster the sequences. Fungal data were analysed using the PIPITS pipeline (Gweon *et al*., 2015). The UNITE database (Abarenkov *et al*., 2010) was used for identification of fungi and the ITSx extractor was used to extract fungal ITS regions (Bengtsson‐Palme *et al*., 2013). FUNGuild (Nguyen *et al*., 2016) was used to classify fungal operational taxonomic units (OTUs) into potential functions and assignment was further curated using an in‐house database (Hannula *et al*., 2017). The OTUs that could be classified were grouped into saprophytes, AMF, potential plant pathogens, plant endophytes and others (ectomycorrhizal fungi, fungal/animal/unidentified plant pathogens). In case of uncertainty on the fungal guild, multiple assignments were allowed (for example all Fusaria were assigned as plant pathogen‐soil saprotroph as the ITS region does not separate for the species in this group). Approximately half of the OTUs making up 46% of reads could be assigned to a specific functional group. The remaining OTUs were assigned to the group ‘unclassified’. All data were used for the analysis, but the subset of classified data was used for the analysis of functional groups. For bacteria, samples with less than 1276 reads or more than 100 000 reads were removed from the dataset, which corresponds roughly to 6 times standard deviation from median. For fungi, the read numbers per sample were standardized against the median sequencing depth (5822 reads) and samples with 3 times higher or lower number of reads than the coefficient variation were removed. This resulted in removal of 14 samples for bacteria and 11 samples for fungi. For bacteria, all reads originating from chloroplasts and mitochondria were removed and for fungi only fungal sequences were used. Furthermore, all OTUs with low total abundance (< 0.001%) and found in less than 4 samples were removed resulting in removal of 11 033 OTUs of bacteria and 786 phylotypes of fungi. The bacterial data were rarified to 1276 reads due to unevensampling depth, high sparsity and complex compositionality. In the nonrarified dataset of bacteria, the read number explained most of the variation in the nonmetric multidimensional scaling (NMDS). We furthermore investigated the effect of rarefaction by using two different thresholds: namely 2527 reads and 5000 reads. With the latter 23 samples and 378 OTUs were removed while for the first 15 samples were removed together with 110 OTUs.

### *Statistical analysis*

Sequencing data were normalized using the total sum scaling (Weiss *et al*., 2017). Rarefaction curves were calculated to investigate the effects of sequencing depth and plant species on the number of OTUs obtained using the package (‘ranacapa’) in R (Kandlikar *et al*., 2018). Effects of plant species identity, plant group (grass, forb or legume) and feedback parameters on the structure of the bacterial and fungal community were then examined using permutational multivariate analysis of variance (PERMANOVA) based on a Bray–Curtis dissimilarity matrix in R [package vegan in R (Oksanen *et al*., 2006)]. Homogeneity in dispersion was investigated using the betadisper function in the package vegan in R (Oksanen *et al*., 2006). For fungi, the legume species *V. sativa* was omitted from further analysis due to too large dispersion in community structure. Separations among treatments were visualized using NMDS of a Bray–Curtis dissimilarity matrix. The effects of plant group and species were further evaluated using PERMANOVA excluding the control samples. Simpson diversity was calculated and the effects of plants species, plant group and feedback parameters on diversity were evaluated using linear regression models for log‐transformed data in R. The abundances or bacterial phyla and relative abundances of fungal phyla, classes and functional groups were evaluated using linear regression models in R using the package nlme (Pinheiro *et al*., 2019). In the models, we used plant group, plants species or one of the chrysanthemum growth parameters as fixed factor, with pot number as a random factor. For the analysis of plant families, plant species was used as a random factor. The normality of distributions was inspected using a Shapiro–Wilk test in R and if assumptions were not met, a Hellinger transformation was used or data were arcsin‐square‐root transformed.

We divided the sequenced soil samples (i.e. the inocula) into response groups based on their effects on Chrysanthemum growth (grasses, forbs and legumes with positive effects, and grasses, forbs and legumes with negative effects). This was done separately for plants that were and were not exposed to the introduced pathogen. The division was not always based on plant species as there was considerable variation in microbiomes and in the effects of soil inoculation within replicates of a single conditioning species. There was a significant effect of category (i.e. grasses, forbs and legumes with positive effects and grasses, forbs and legumes with negative effects) on the biomass of Chrysanthemum grown in the soils conditioned with the selected plants (see Supporting Information Fig. S2). The distances of fungal and bacterial communities in individual plants were calculated using Bray–Curtis distance with 999 permutations in the package Vegan in R. Dendrograms were created using these distances and Ward's D was used to cluster the samples.

## Conclusions

In conclusion, we show that effects of plants both on the soil microbiome and on the growth of chrysanthemum (through those effects on the microbiome), are plant species‐specific and not strongly conserved at the level of plant family or among grasses, forbs or legumes. This is in line with previous work showing that broad plant groups affect the soil through different mechanisms (Pérez‐Jaramillo *et al*., 2018). Most of the grasses that we tested show a positive feedback effect on the growth of Chrysanthemum (Ma *et al*., 2017) but we urge that this is not a general effect of all grass species as some grass species caused negative PSF effect and some forb species had positive effects on the growth of Chrysanthemum. We argue that we should select suitable plants to be used to create positive PSFs based on their microbiomes and not based on the family or broad group the plant belongs to. Plants shaping their bacterial community structure into types B1 and B2 and fungal type F1 in our study had the most beneficial effect on the growth of the following plant in the presence of a pathogen. Further studies should examine what characteristics of their impact on the microbiome make these species cluster together, and whether these species also positively influence other crops.

## Funding

This study was funded by the Netherlands Organization for Scientific Research NWO VICI grant 865.14.006 and NWO Groen, project no. 870.15.080. H.M. was funded by the Chinese Scholarship Council (CSC).

## Author Contributions

The experiment was designed by T.M.B. and H.M. H.M. performed the greenhouse experiments. S.E.H., T.M.B., A.P., H.M., J.E.P.‐J. discussed the data analysis. J.E.P.‐J. and S.E.H. performed bioinformatics analysis and S.E.H. performed all statistical analysis. S.E.H. wrote the first draft of the manuscript in collaboration with T.M.B. and H.M. A.P. and J.E.P.‐J. commented on the manuscript and all authors approved the final manuscript.

## Conflict of Interest

The authors declare that they have no competing interests.

## Supporting information


**Data S1**: Supporting Information

**Data S2**: Supporting Information

## Data Availability

The datasets generated and analysed during the current study are available in the ENA repository under accession number PRJEB31284. OTU tables are included as Supporting Information.
