# Supplementary material for: Structure and ecological function of the soil microbiome affecting plant–soil feedbacks in the presence of a soil‐borne pathogen
Source: Environ Microbiol. 2019 Dec 18;22(2):660–76. doi: 10.1111/1462-2920.14882 (PMC7027455; doi:10.1111/1462-2920.14882)
Supplement: Supplementary file 1 — Data S1: Supporting Information [file EMI-22-660-s001.docx]

These are supplementary figures and tables of:

**Structure and ecological function of the soil microbiome affecting plant-soil feedbacks in the presence of a soil-borne pathogen**

Hannula, S.E., Ma, H., Pérez-Jaramillo J.E., Pineda, A. & Bezemer, T.M.


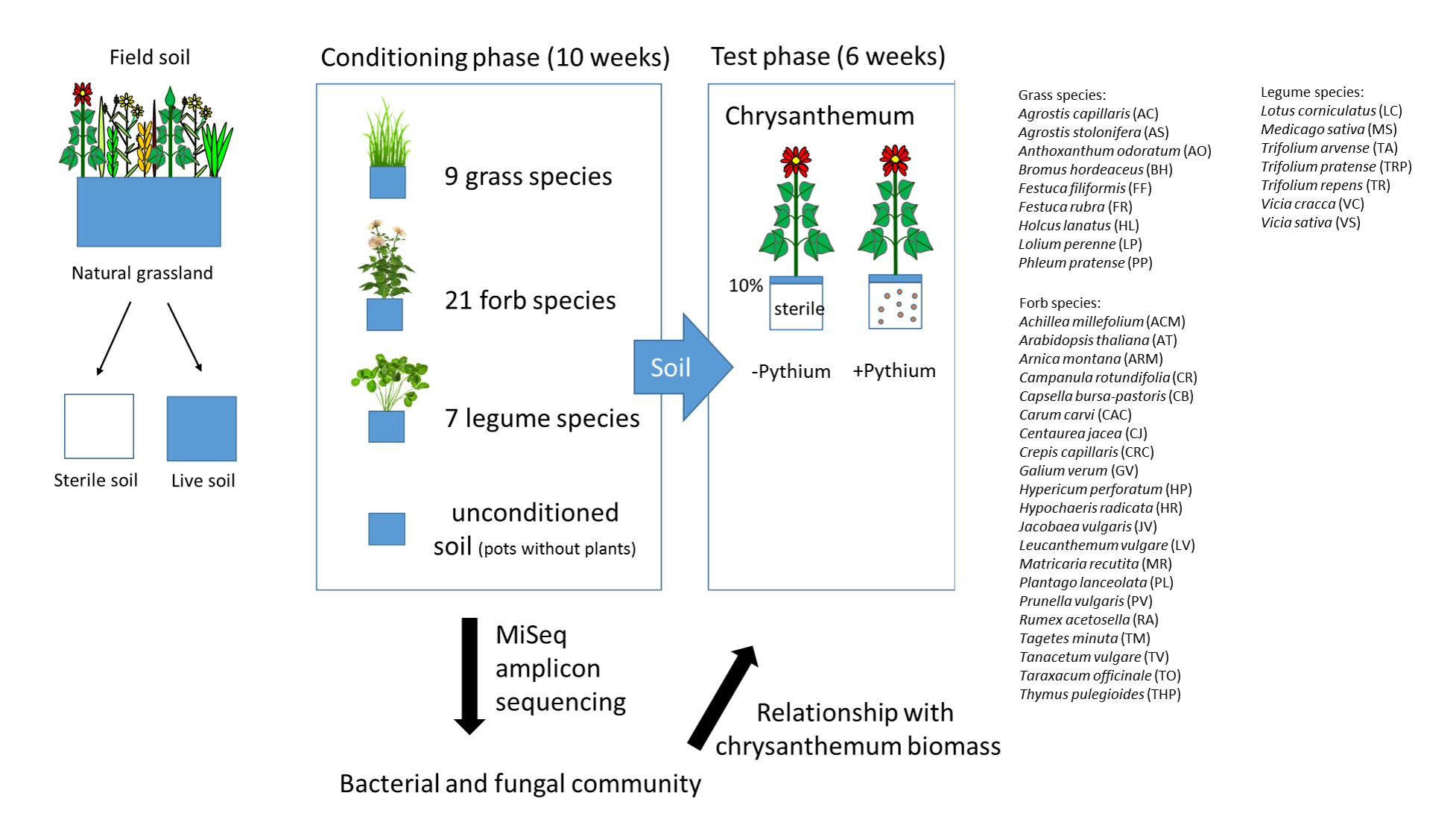


**Supplementary figure 1**. Schematic overview of the experiment. The plant species used to condition the soil are also presented (codes in brackets).


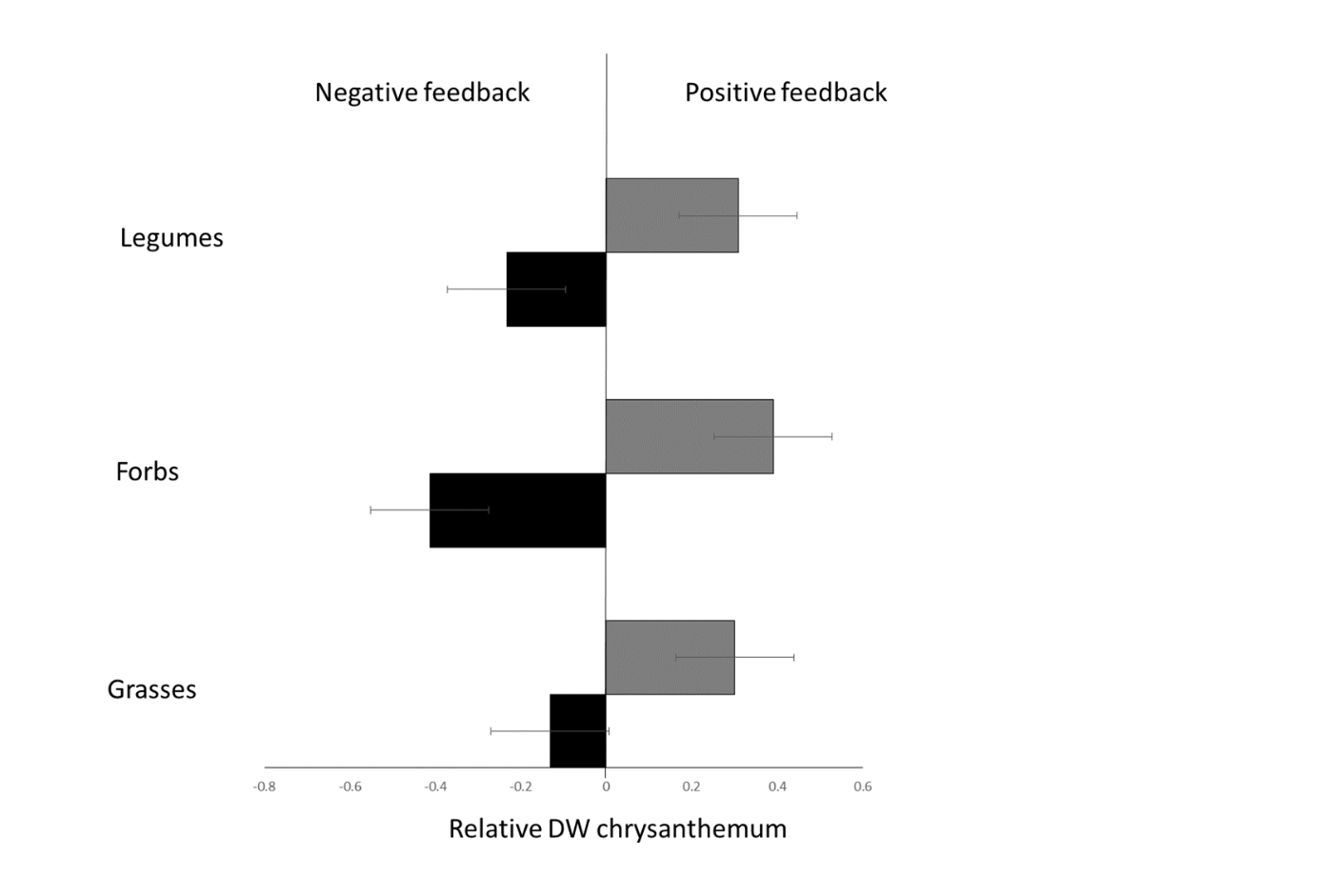


**Supplementary figure 2.** The average relative dry weight (DW) of chrysanthemum in the six categories artificially created (‘legumes causing negative feedback’, ‘legumes causing positive feedback’, ‘forbs causing negative feedback, forbs causing positive feedback’, ‘grasses causing negative feedback’, grasses causing positive feedback’). The error bars depict standard error of the categories. Total number of plants included in this analysis was 78, of which 16 are legumes, 18 are grasses and 44 are forbs, half having positive and half negative feedback.

**
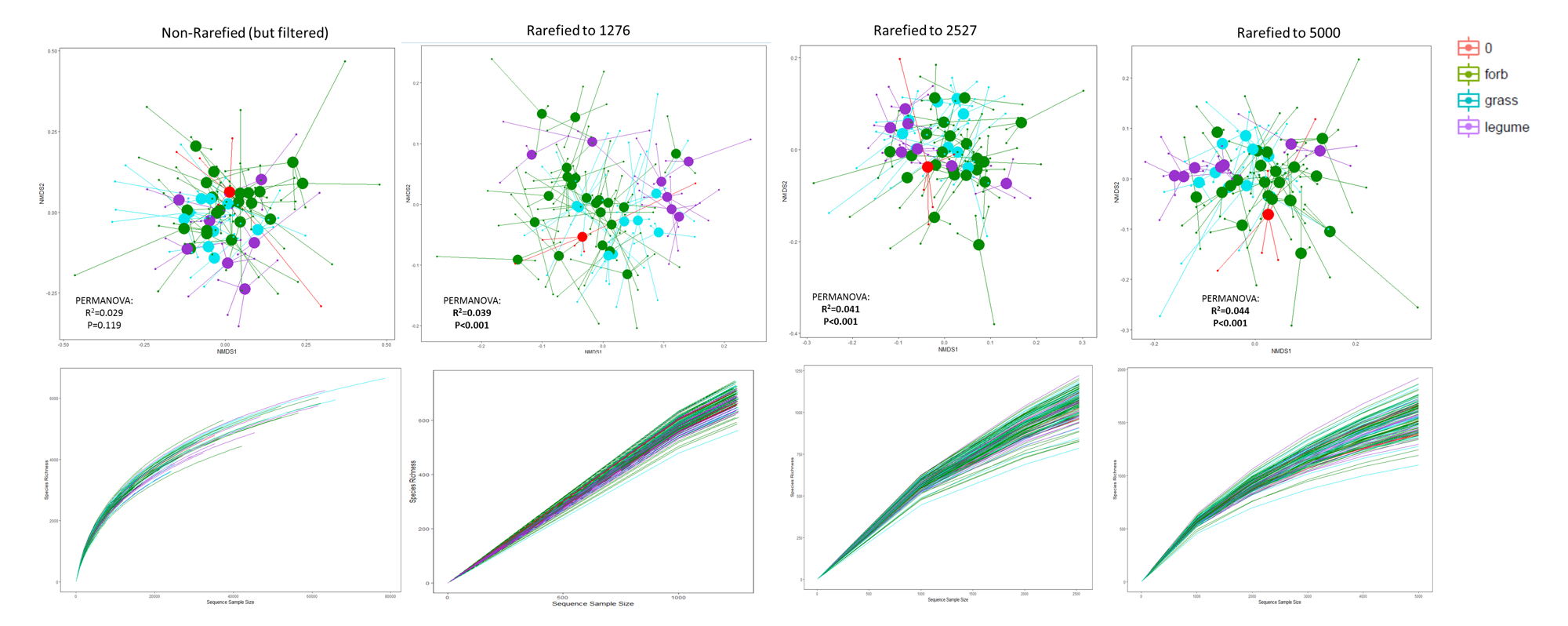
Supplementary figure 3.** Effects of different levels of rarefaction on the community structure of bacteria. Colors separate soils from grass species, forb species, legume species, and no-plant soil (0). Results from PERMANOVA for plant group are shown for each rarefication option.


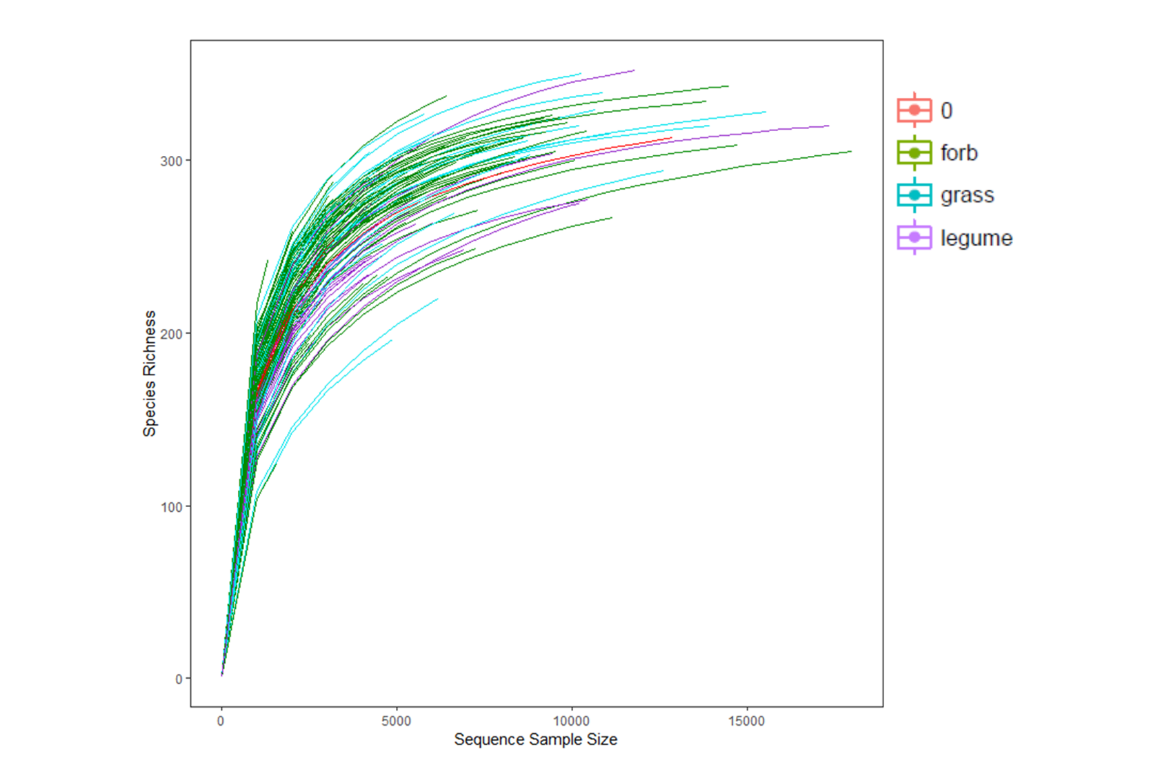


**Supplementary figure 4.** Rarefaction curve for fungi. Colors separate soils from grass species, forb species, legume species, and no-plant soil (0).


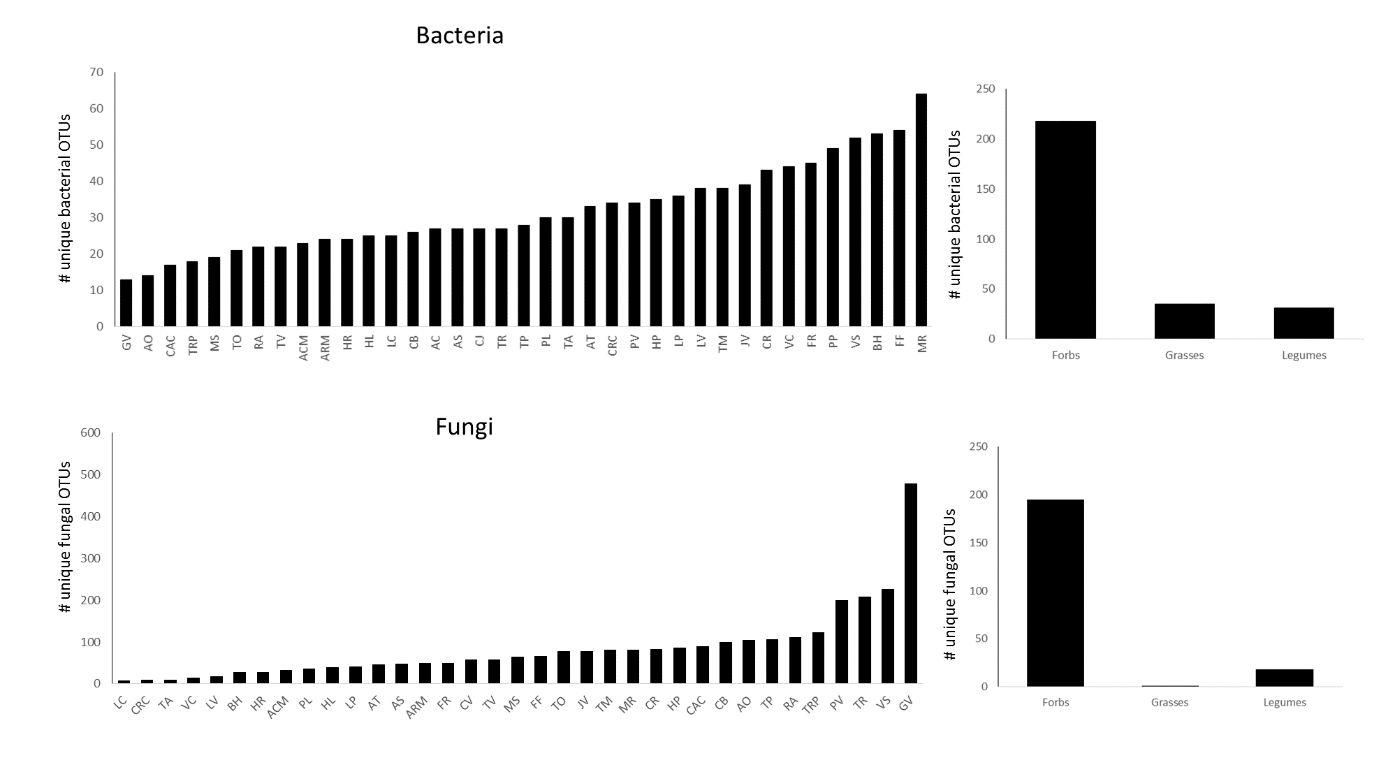


**Supplementary figure 5.** Numbers of unique bacterial and fungal OTUs per plant species and for the three plant groups. The plant names corresponding to the codes used here can be found in supplementary figure 1.

**
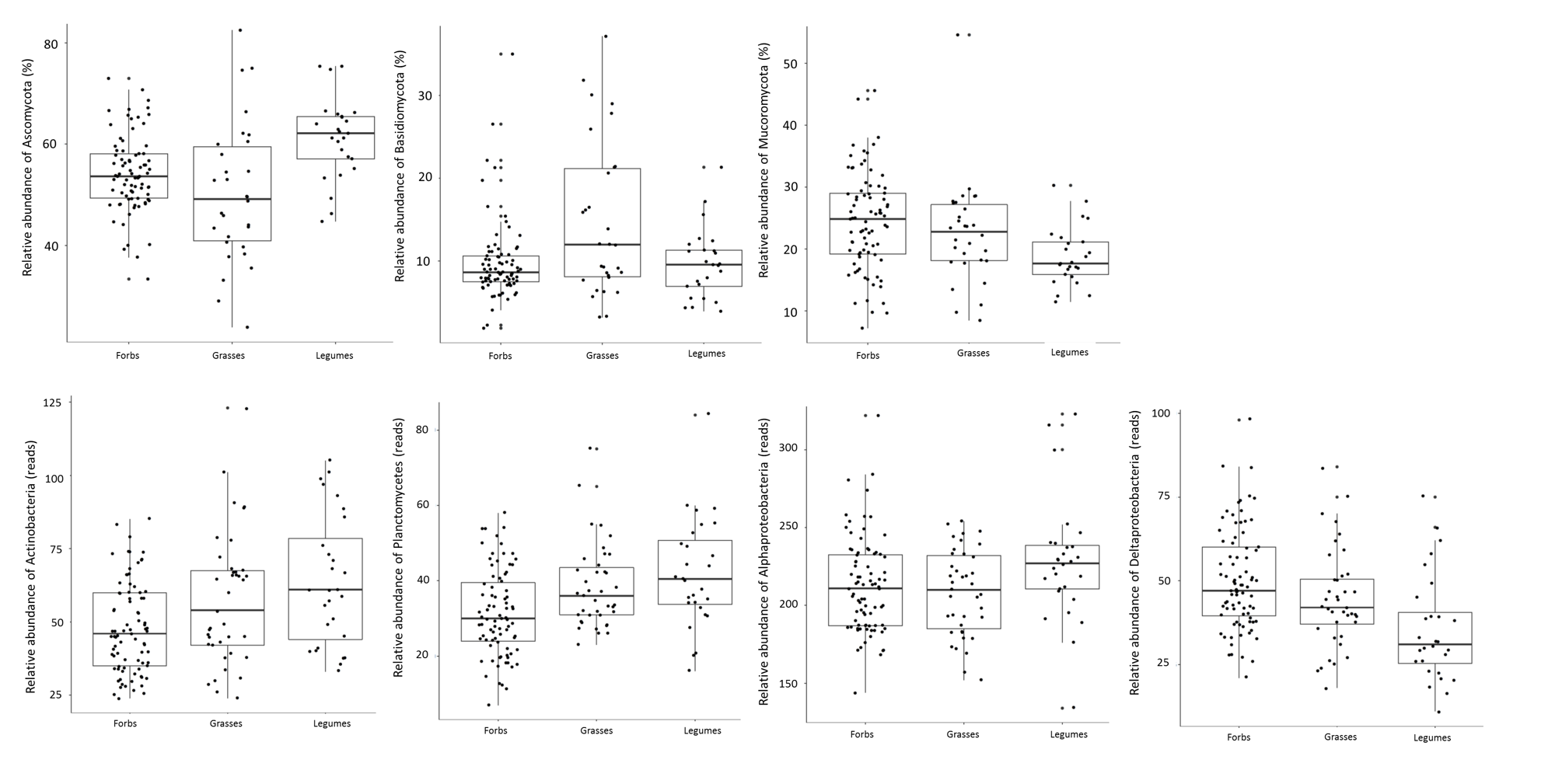
Supplementary figure 6.** Relative abundances of fungal and bacterial phyla per plant group. For statistical significance see table 2. Box-and-whisker plots report median relative abundances and whiskers and scatter full variance in abundances of fungal and bacterial phyla per plant functional category.

**
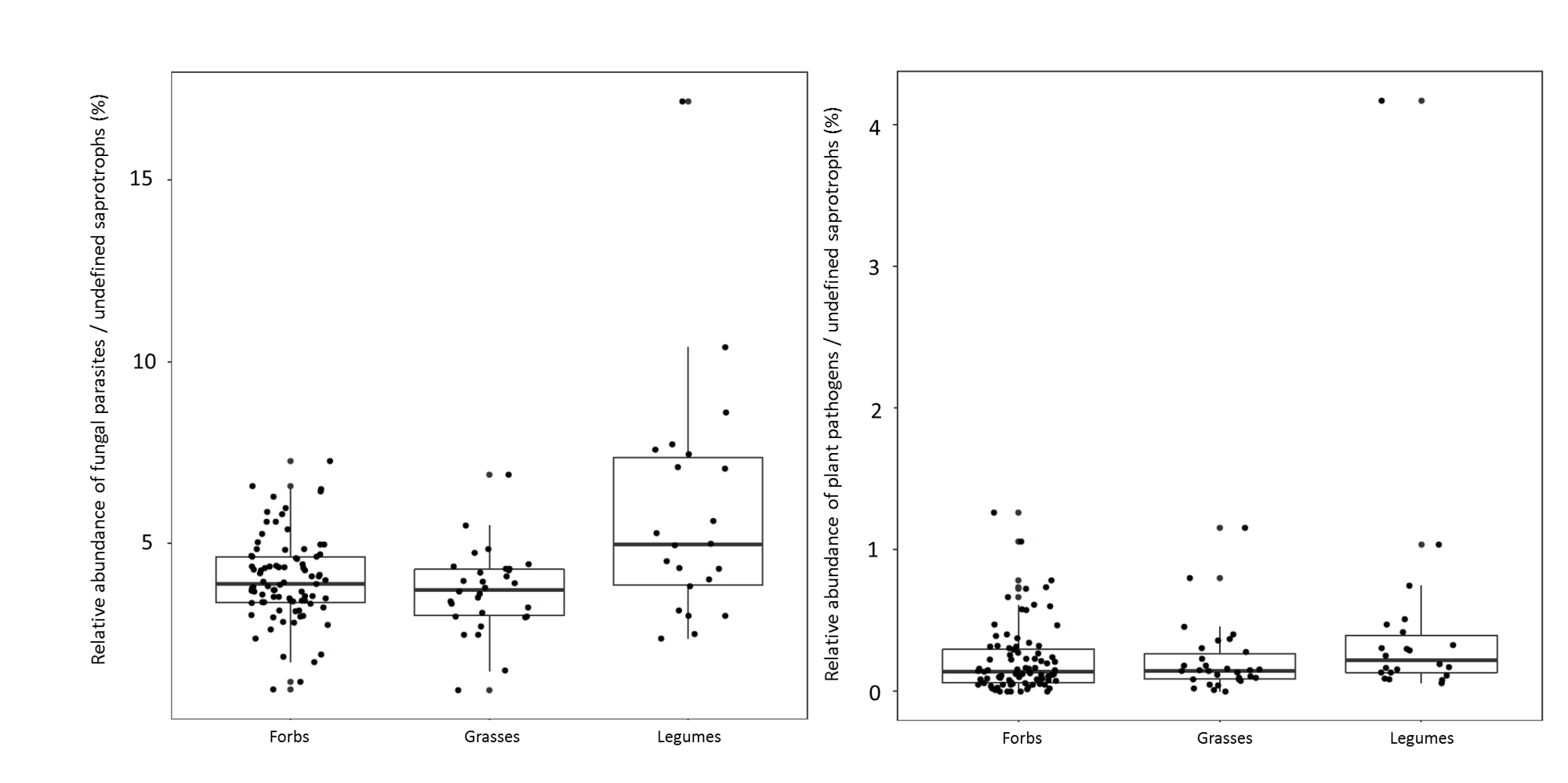
Supplementary figure 7** Relative abundances of fungal guilds per plant group. For statistical significance see supplementary table 2. Box-and-whisker plots report median relative abundances and whiskers and scatter full variance in relative abundances of fungal functional groups per plant functional category.


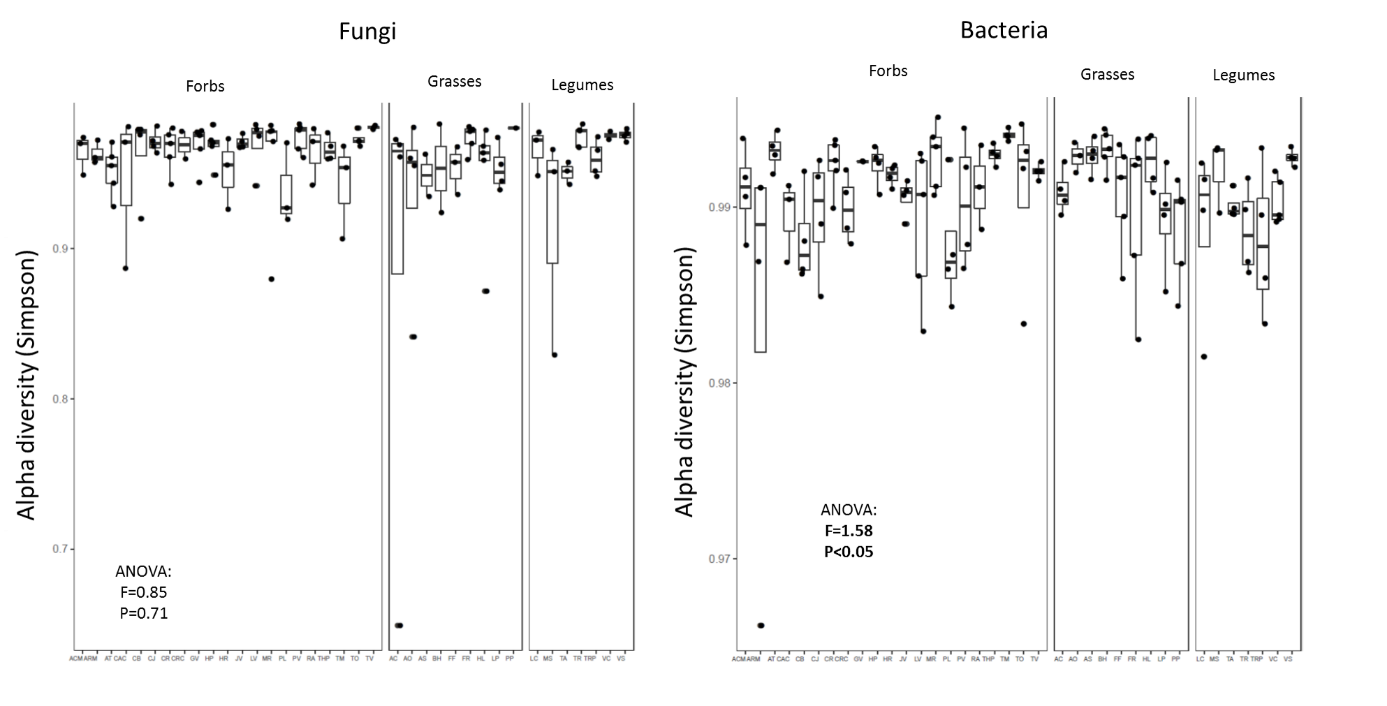


**Supplementary figure 8.** The diversity measured with Simpson index per individual plant species for fungi and bacteria. Significance from ANOVA test are given in the figure. Box-and-whisker plots report median and whiskers and scatter variation in diversity per plant. The plant names corresponding to the codes used here can be found in supplementary table 1.


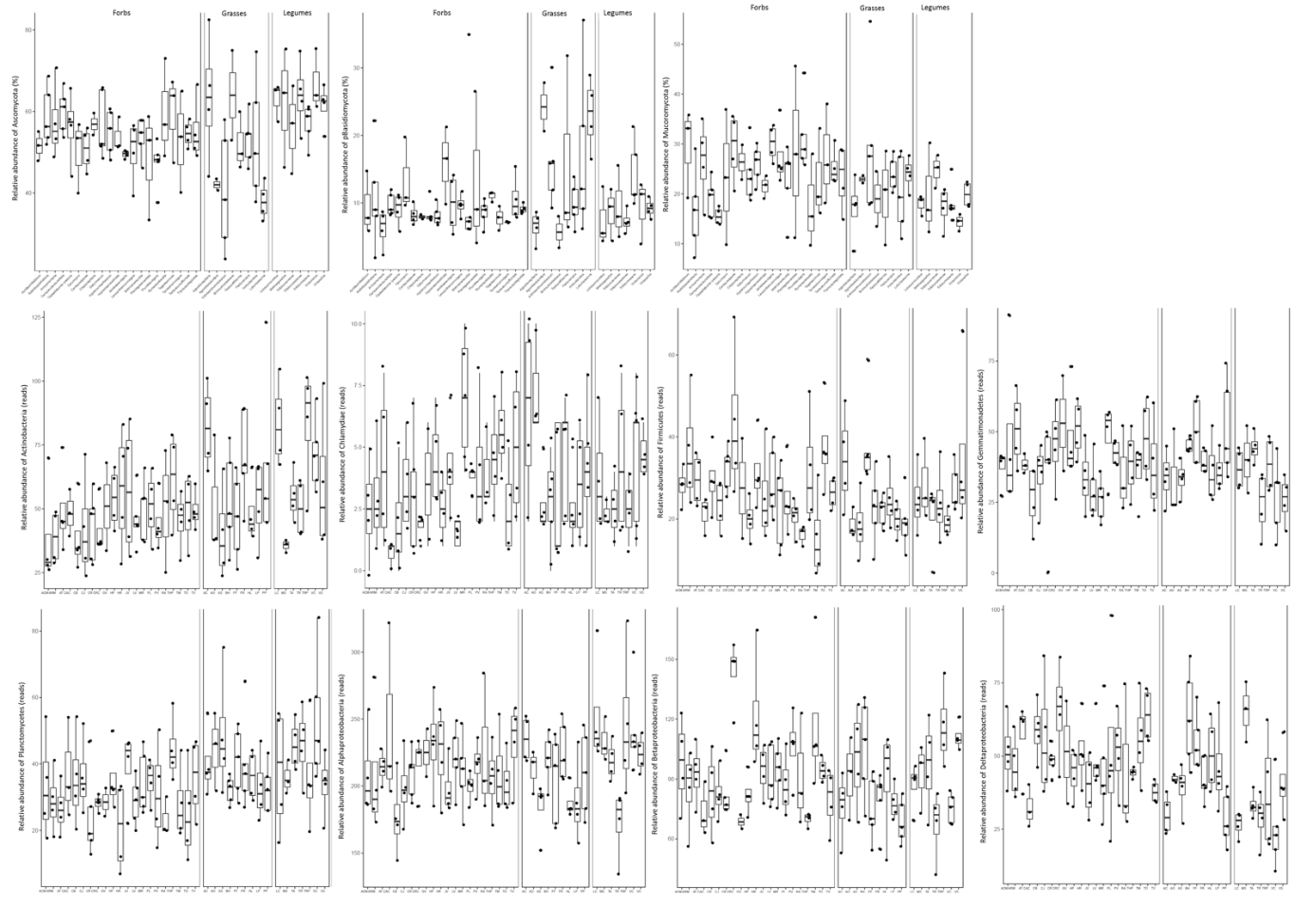


**Supplementary figure 9.** Relative abundances of fungal and bacterial phyla per plant species. For statistical significance see table 2. Box-and-whisker plots report median and whiskers and scatter full variance in abundances of fungal and bacterial phyla per plant species. The plant names corresponding to the codes used here can be found in supplementary table 1.


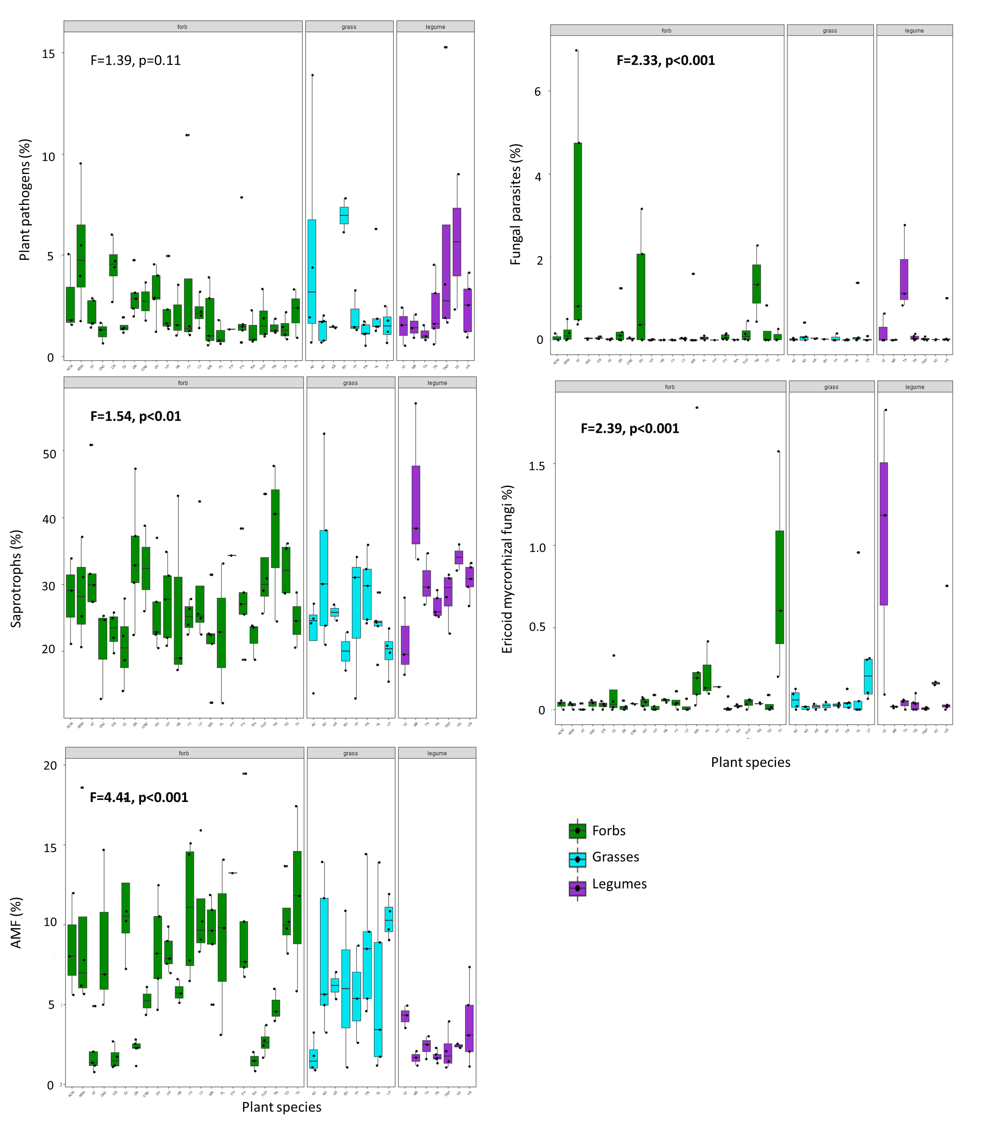


**Supplementary figure 10.** Relative abundances of fungal functional guilds per plant species. For statistical significance see supplementary table 2. Box-and-whisker plots report median relative abundances and whiskers and scatter full variance in relative abundances of fungal functional groups per plant species. The plant names corresponding to the codes used here can be found in supplementary table 1.


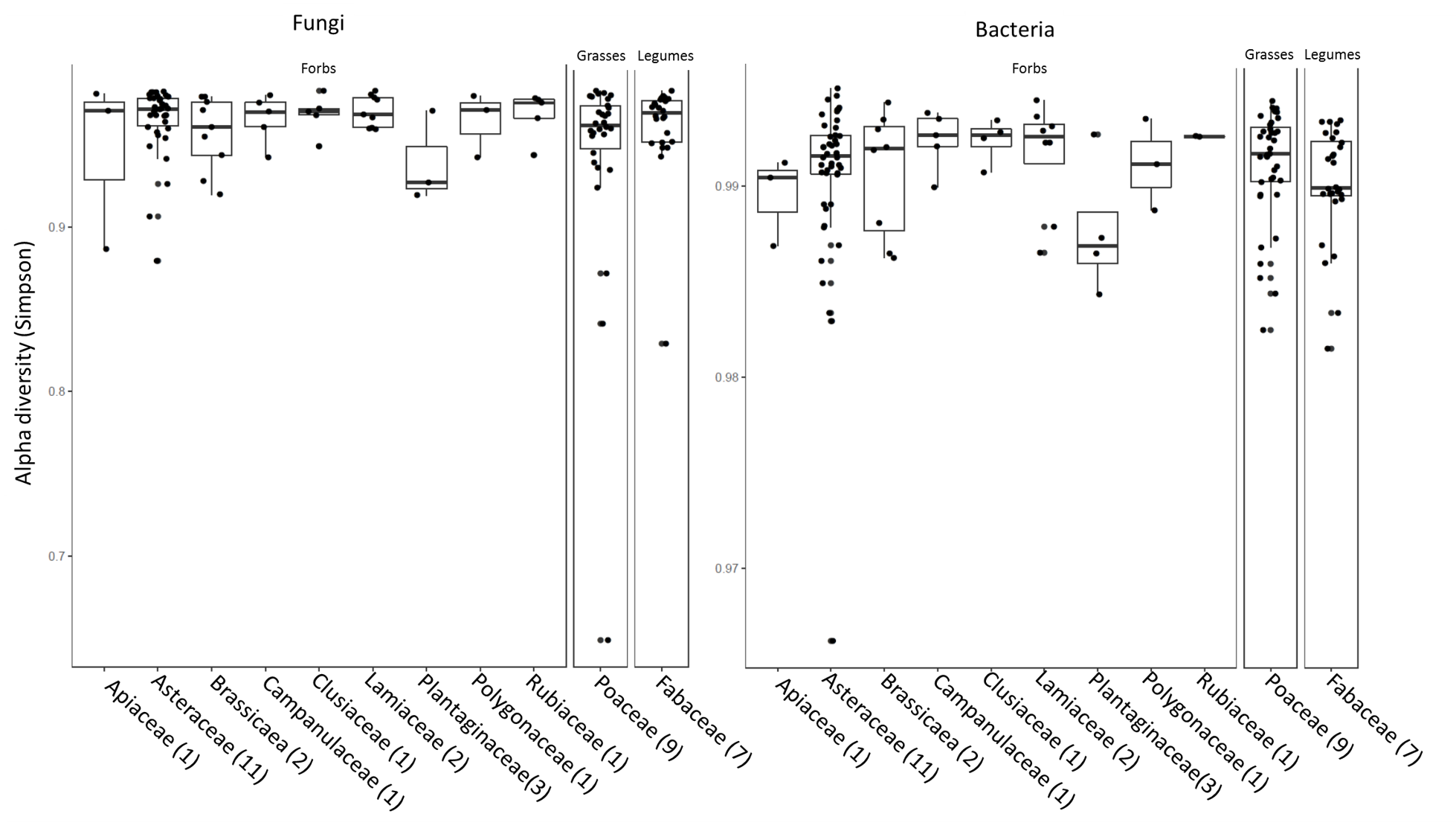


**Supplementary figure 11.** Diversity (Simpson index) per plant family for fungi and bacteria. Box-and-whisker plots report median and whiskers and scatter variation in diversity per plant family. Plot is divided into groups based on plant functional group. Statistical results are presented in Table 1.


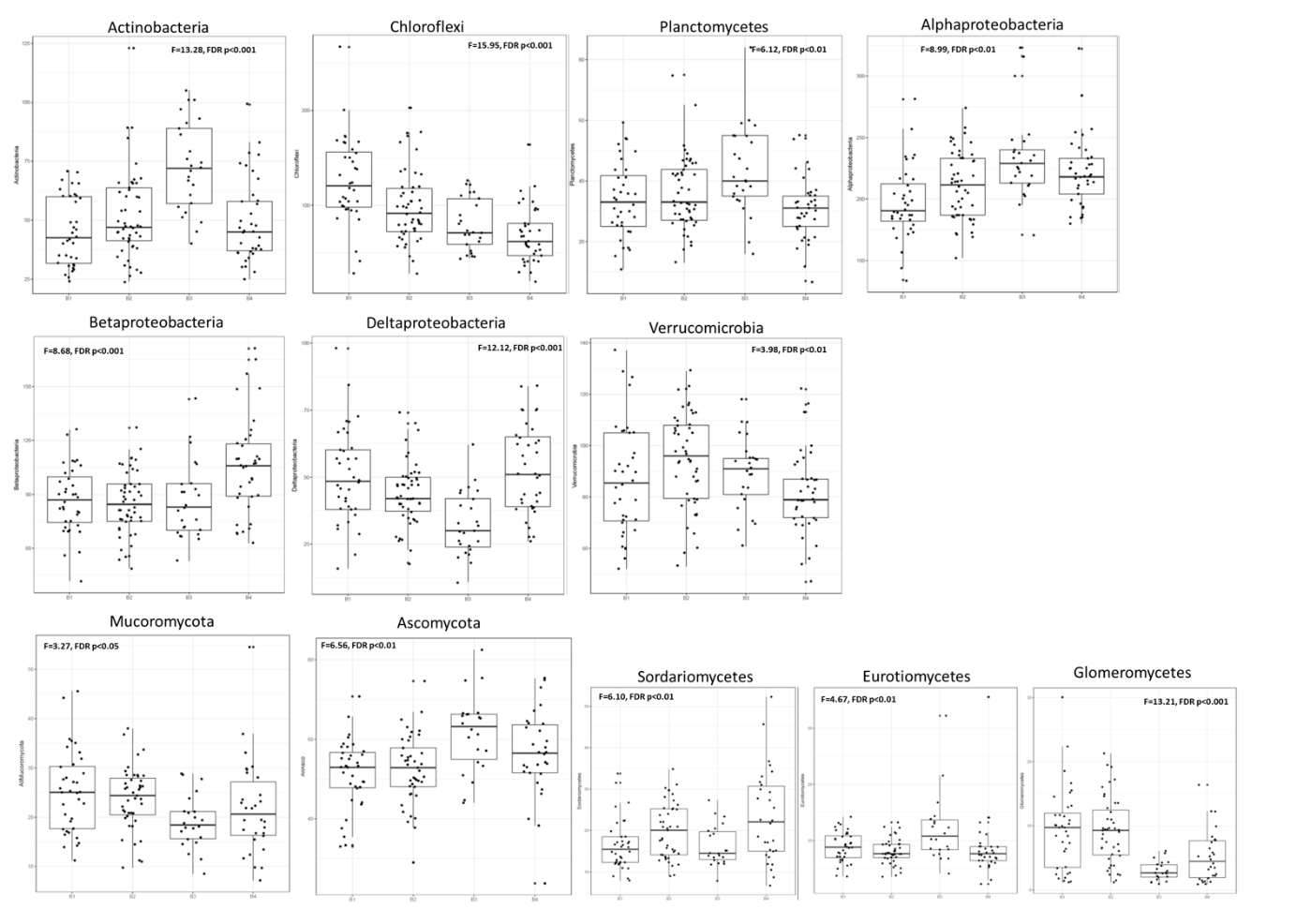


**Supplementary figure 12**. Bacterial phyla and fungal phyla and classes that are significantly different in relative abundance in the clusters presented in figure 5. Box-and-whisker plots report median and whiskers and scatter variation in abundance of phyla and classes.

**Suppl. table 1.** Potential effects of the plant species used in the conditioning stage on the soil.

| Species | Group | Soil effects | Reference |
| --- | --- | --- | --- |
| *Agrostis capillaris* (AC) | Grass | increases soil nitrification rates;  weak association with mycorrhizal fungi | 61; 62 |
| *Agrostis stolonifera* (AS) | Grass |  |  |
| *Anthoxanthum odoratum* (AO) | Grass | increases the abundance of mycorrhizal fungi;  increases soil microbial biomass and respiration | 63; 64 |
| *Bromus hordeaceus* (BH) | Grass | higher nitrogen flow due to mycorrhizal fungi | 65; 66 |
| *Festuca filiformis* (FF) | Grass |  |  |
| *Festuca rubra* (FR) | Grass | Suppression of a plant parasitic nematode | 67 |
| *Holcus lanatus* (HL) | Grass | increases microbial biomass and respiration;  allocates more carbon to bacteria; increases Actinomycetes;  promotes a bacteria-dominated soil food web;  reduces the number of bacteria-feeding nematodes | 64; 68; 69; 70 |
| *Lolium perenne* (LP) | Grass | increases the abundance of antibiotic-producing bacteria;  increases soil nitrification rates; | 71;62; 72; 73 |
| *Phleum pratense* (PP) | Grass |  |  |
| *Achillea millefolium* (ACM) | Forb | sensitive to soil fungi | 74; 75 |
| *Arabidopsis thaliana* (AT) | Forb | model plant, non-mycorrhizal | 76; 77 |
| *Arnica montana* (ARM) | Forb | strong dependence on mycorrhizal fungi | 78; 79 |
| *Campanula rotundifolia* (CR) | Forb | strong dependence on mycorrhizal fungi | 80 |
| *Capsella bursa-pastoris* (CB) | Forb |  |  |
| *Carum carvi* (CAC) | Forb |  |  |
| *Centaurea jacea* (CJ) | Forb |  |  |
| *Crepis capillaris* (CRC) | Forb |  |  |
| *Hypericum perforatum* (HP) | Forb |  |  |
| *Hypochaeris radicata* (HR) | Forb |  |  |
| *Jacobaea vulgaris* (JV) | Forb | chemical compounds extracted from roots have inhibitory effects on plant-associated fungi | 82 |
| *Leucanthemum vulgare* (LV) | Forb | increases the abundance of mycorrhizal fungi | 83; 84 |
| *Matricaria recutita* (MR) | Forb |  |  |
| *Plantago lanceolata* (PL) | Forb | increases the abundance of mycorrhizal fungi;  inhibitory effect on soil N mineralization | 85; 86; 81 |
| *Prunella vulgaris* (PV) | Forb | increases the abundance of mycorrhizal fungi | 61 |
| *Rumex acetosella* (RA) | Forb | low abundance of bacteria in its root | 87 |
| *Tagetes minuta* (TM) | Forb;  A garden plant | antagonisitic effect on plant parasitic nematodes | 88; 89; 90; 91 |
| *Tanacetum vulgare* (TV) | Forb | antimicrobial effects of root extracts | 92 |
| *Taraxacum officinale* (TO) | Forb | reduces the diversity of non-mycorrhizal fungi;  increases AMF abundance; improves soil aggregation | 93; 94 |
| *Thymus pulegioides* (THP) | forb |  |  |
| *Lotus corniculatus* (LC) | Legume | fungi-dominated soil web;  reduces the diversity of soil microbes;  increases soil carbon and nitrogen stocks | 69; 95; 96 |
| *Medicago sativa* (MS) | Legume | increases the nitrogen uptake of later crop;  increases the abundance of soil bacteria and fungi | 97; 98; 99 |
| *Trifolium arvense* (TA) | Legume |  |  |
| *Trifolium pratense* (TRP) | Legume | increases the nitrogen uptake of later crop;  increases the abundance of mycorrhizal fungi | 97; 100 |
| *Trifolium repens* (TR) | Legume | increases soil carbon and nitrogen stocks;  increases the nitrogen uptake of later crop;  increases the diversity of *Pseudomonas*;  increases the diversity of soil bacterial and fungal communities | 96; 101; 102; 97 |
| *Vicia cracca* (VC) | Legume |  |  |
| *Vicia sativa* (VS) | Legume | suppresses plant parasitic nematodes;  increases soil nitrogen; increases soil carbon liability | 103; 104; 105 |

**Suppl. table 2.** The effects of plant species and plant group (grasses, forbs, or legumes) on the relative abundances of fungal guilds and the relationship between the abundance of these guilds and the growth of Chrysanthemum in the presence and absence of the pathogen *Pythium*. Significant values derived from LME model are marked in bold.

References accompanying supplementary figures and tables:

61. Veresoglou SD, Sen R, Mamolos AP, Veresoglou DS: **Plant species identity and arbuscular mycorrhizal status modulate potential nitrification rates in nitrogen‐limited grassland soils**. *Journal of Ecology* 2011, **99**(6):1339-1349.

62. Weigelt A, King R, Bol R, Bardgett RD: **Inter‐specific variability in organic nitrogen uptake of three temperate grassland species**. *J Plant Nutr Soil Sc* 2003, **166**(5):606-611.

63. De Deyn GB, Quirk H, Bardgett RD: **Plant species richness, identity and productivity differentially influence key groups of microbes in grassland soils of contrasting fertility**. *Biology letters* 2010, **7**(1):75-78.

64. Innes L, Hobbs PJ, Bardgett RD: **The impacts of individual plant species on rhizosphere microbial communities in soils of different fertility**. *Biology and Fertility of Soils* 2004, **40**(1):7-13.

65. Costello MJ: **Growth and yield of cultivated grape with native perennial grasses nodding needlegrass or California barley as cover crops**. *Hortscience* 2010, **45**(1):154-156.

66. Cheng X, Baumgartner K: **Arbuscular mycorrhizal fungi-mediated nitrogen transfer from vineyard cover crops to grapevines**. *Biology and fertility of soils* 2004, **40**(6):406-412.

67. Vrain T, DeYoung R, Hall J, Freyman S: **Cover crops resistant to root-lesion nematodes in raspberry**. *Hortscience* 1996, **31**(7):1195-1198.

68. Ladygina N, Hedlund K: **Plant species influence microbial diversity and carbon allocation in the rhizosphere**. *Soil Biology and Biochemistry* 2010, **42**(2):162-168.

69. Witt C, Setälä H: **Do plant species of different resource qualities form dissimilar energy channels below-ground?** *Applied soil ecology* 2010, **44**(3):270-278.

70. Saj S, Mikola J, Ekelund F: **Species-specific effects of live roots and shoot litter on soil decomposer abundances do not forecast plant litter-nitrogen uptake**. *Oecologia* 2009, **161**(2):331-341.

71. Latz E, Eisenhauer N, Scheu S, Jousset A: **Plant identity drives the expression of biocontrol factors in a rhizosphere bacterium across a plant diversity gradient**. *Functional Ecology* 2015, **29**(9):1225-1234.

72. Brant V, Neckář K, Pivec J, Duchoslav M, Holec J, Fuksa P, Venclová V: **Competition of some summer catch crops and volunteer cereals in the areas with limited precipitation**. *Plant, Soil and Environment* 2009, **55**(1):17-24.

73. Mauro RP, Anastasi U, Lombardo S, Pandino G, Pesce R, Alessia R, Mauromicale G: **Cover crops for managing weeds, soil chemical fertility and nutritional status of organically grown orange orchard in Sicily**. *Italian Journal of Agronomy* 2015, **10**(2):101-104.

74. Dastgheib F, Kumar K, Goh KM: **Weed infestations in wheat cropping systems as affected by crop residues and their management practices**. *Biol Agric Hortic* 1999, **16**(4):395-407.

75. Šmilauer P, Šmilauerová M: **Effect of AM symbiosis exclusion on grassland community composition**. *Folia Geobot* 2000, **35**(1):13-25.

76. Huot B, Yao J, Montgomery BL, He SY: **Growth–defense tradeoffs in plants: a balancing act to optimize fitness**. *Molecular plant* 2014, **7**(8):1267-1287.

77. Initiative AG: **Analysis of the genome sequence of the flowering plant Arabidopsis thaliana**. *nature* 2000, **408**(6814):796.

78. Jurkiewicz A, Ryszka P, Anielska T, Waligórski P, Białońska D, Góralska K, Tsimilli-Michael M, Turnau K: **Optimization of culture conditions of Arnica montana L.: effects of mycorrhizal fungi and competing plants**. *Mycorrhiza* 2010, **20**(5):293-306.

79. Wardecki T, Brötz E, De Ford C, von Loewenich FD, Rebets Y, Tokovenko B, Luzhetskyy A, Merfort I: **Endophytic Streptomyces in the traditional medicinal plant Arnica montana L.: secondary metabolites and biological activity**. *Antonie van Leeuwenhoek* 2015, **108**(2):391-402.

80. Stevens CJ, Wilson J, McAllister HA: **Biological flora of the British Isles: Campanula rotundifolia**. *Journal of Ecology* 2012, **100**(3):821-839.

81. Miglécz T, Valkó O, Török P, Deák B, Kelemen A, Donkó Á, Drexler D, Tóthmérész B: **Establishment of three cover crop mixtures in vineyards**. *Scientia Horticulturae* 2015, **197**:117-123.

82. Hol W, Van Veen J: **Pyrrolizidine alkaloids from Senecio jacobaea affect fungal growth**. *Journal of Chemical Ecology* 2002, **28**(9):1763-1772.

83. Reidinger S, Eschen R, Gange AC, Finch P, Bezemer TM: **Arbuscular mycorrhizal colonization, plant chemistry, and aboveground herbivory on Senecio jacobaea**. *Acta oecologica* 2012, **38**:8-16.

84. Bharadwaj DP, Lundquist P-O, Alström S: **Impact of plant species grown as monocultures on sporulation and root colonization by native arbuscular mycorrhizal fungi in potato**. *Applied soil ecology* 2007, **35**(1):213-225.

85. Šmilauer P: **Communities of arbuscular mycorrhizal fungi in grassland: seasonal variability and effects of environment and host plants**. *Folia Geobot* 2001, **36**(3):243-263.

86. Dietz M, Machill S, Hoffmann HC, Schmidtke K: **Inhibitory effects of Plantago lanceolata L. on soil N mineralization**. *Plant and Soil* 2013, **368**(1-2):445-458.

87. Vale M, Nguyen C, Dambrine E, Dupouey J: **Microbial activity in the rhizosphere soil of six herbaceous species cultivated in a greenhouse is correlated with shoot biomass and root C concentrations**. *Soil Biology and Biochemistry* 2005, **37**(12):2329-2333.

88. Hooks CR, Wang K-H, Ploeg A, McSorley R: **Using marigold (Tagetes spp.) as a cover crop to protect crops from plant-parasitic nematodes**. *Applied Soil Ecology* 2010, **46**(3):307-320.

89. Kimpinski J, Arsenault W, Gallant C, Sanderson J: **The effect of marigolds (Tagetes spp.) and other cover crops on Pratylenchus penetrans and on following potato crops**. *J Nematol* 2000, **32**(4S):531.

90. Natarajan N, Cork A, Boomathi N, Pandi R, Velavan S, Dhakshnamoorthy G: **Cold aqueous extracts of African marigold, Tagetes erecta for control tomato root knot nematode, Meloidogyne incognita**. *Crop Protection* 2006, **25**(11):1210-1213.

91. Sturz A, Kimpinski J: **Endoroot bacteria derived from marigolds (Tagetes spp.) can decrease soil population densities of root-lesion nematodes in the potato root zone**. *Plant and Soil* 2004, **262**(1-2):241-249.

92. Devrnja N, Anđelković B, Aranđelović S, Radulović S, Soković M, Krstić-Milošević D, Ristić M, Ćalić D: **Comparative studies on the antimicrobial and cytotoxic activities of Tanacetum vulgare L. essential oil and methanol extracts**. *South African Journal of Botany* 2017, **111**:212-221.

93. Becklin KM, Hertweck KL, Jumpponen A: **Host identity impacts rhizosphere fungal communities associated with three alpine plant species**. *Microb Ecol* 2012, **63**(3):682-693.

94. Kabir Z, Koide R: **The effect of dandelion or a cover crop on mycorrhiza inoculum potential, soil aggregation and yield of maize**. *Agriculture, ecosystems & environment* 2000, **78**(2):167-174.

95. Li H, Cheng Z: **Hoagland nutrient solution promotes the growth of cucumber seedlings under light-emitting diode light**. *Acta Agriculturae Scandinavica, Section B—Soil & Plant Science* 2015, **65**(1):74-82.

96. De Deyn GB, Quirk H, Yi Z, Oakley S, Ostle NJ, Bardgett RD: **Vegetation composition promotes carbon and nitrogen storage in model grassland communities of contrasting soil fertility**. *Journal of Ecology* 2009, **97**(5):864-875.

97. Amossé C, Jeuffroy M-H, Mary B, David C: **Contribution of relay intercropping with legume cover crops on nitrogen dynamics in organic grain systems**. *Nutrient Cycling in Agroecosystems* 2014, **98**(1):1-14.

98. Chang C, Zhou X, Fu X, Yang S, Wu F: **Soil enzymes and bacterial community composition in cucumber (Cucumis sativus L.) monocropping and companion cropping systems**. *Allelopathy Journal* 2016, **38**(2):133-146.

99. Zhao J, Zeng Z, He X, Chen H, Wang K: **Effects of monoculture and mixed culture of grass and legume forage species on soil microbial community structure under different levels of nitrogen fertilization**. *Eur J Soil Biol* 2015, **68**:61-68.

100. Veiga RS, Faccio A, Genre A, Pieterse CM, Bonfante P, van der Heijden MG: **Arbuscular mycorrhizal fungi reduce growth and infect roots of the non‐host plant *Arabidopsis thaliana***. *Plant, cell & environment* 2013, **36**:1926-1937.

101. Chang C-l, Fu X-p, Zhou X-g, Gup M-y, Wu F-z: **Effects of seven different companion plants on cucumber productivity, soil chemical characteristics and Pseudomonas community**. *Journal of Integrative Agriculture* 2017, **16**:2206-2214.

102. Li S, Wu F: **Diversity and cooccurrence patterns of soil bacterial and fungal communities in seven intercropping systems**. *Frontiers in Microbiology* 2018, **9**:1521.

103. Bayer C, Dieckow J, Amado TJC, Eltz FLF, Vieira FCB: **Cover crop effects increasing carbon storage in a subtropical no‐till sandy Acrisol**. *Commun Soil Sci Plan* 2009, **40**:1499-1511.

104. Novara A, Gristina L, Guaitoli F, Santoro A, Cerdà A: **Managing soil nitrate with cover crops and buffer strips in Sicilian vineyards**. *Solid Earth* 2013, **4**(2):255-262.

105. Hagan A, Gazaway W, Sikora E: **Nematode suppressive crops**. *The Alabama Cooperative Extension System. Sikora, RA (ed) Alabama A&M University and Auburn University, ANR* 1998, 856.
